# Differentiation of human ESCs to retinal ganglion cells using a CRISPR engineered reporter cell line

**DOI:** 10.1038/srep16595

**Published:** 2015-11-13

**Authors:** Valentin M. Sluch, Chung-ha O. Davis, Vinod Ranganathan, Justin M. Kerr, Kellin Krick, Russ Martin, Cynthia A. Berlinicke, Nicholas Marsh-Armstrong, Jeffrey S. Diamond, Hai-Quan Mao, Donald J. Zack

**Affiliations:** 1 Department of Molecular Biology and Genetics, Johns Hopkins University School of Medicine Baltimore, MD 21287; 2 Hugo W. Moser Research Institute, Kennedy Krieger Institute, Baltimore, MD, 21205; 3 Department of Ophthalmology, Wilmer Eye Institute, Johns Hopkins University School of Medicine, Baltimore, MD 21287; 4 Synaptic Physiology Section, National Institute of Neurological Disorders and Stroke, National Institutes of Health, Bethesda, MD 20892; 5 Department of Biomedical Engineering, Johns Hopkins University School of Medicine, Baltimore, MD 21205; 6 Translational Tissue Engineering Center, Johns Hopkins University School of Medicine, Baltimore, MD 21287; 7 Department of Materials Science and Engineering, Whiting School of Engineering, and Institute for NanoBioTechnology, Johns Hopkins University, Baltimore, MD 21218; 8 The Solomon H. Snyder Department of Neuroscience, Johns Hopkins University School of Medicine, Baltimore, MD, 21205; 9 Institute of Genetic Medicine, Johns Hopkins University School of Medicine, Baltimore, MD 21287.

## Abstract

Retinal ganglion cell (RGC) injury and cell death from glaucoma and other forms of optic nerve disease is a major cause of irreversible vision loss and blindness. Human pluripotent stem cell (hPSC)-derived RGCs could provide a source of cells for the development of novel therapeutic molecules as well as for potential cell-based therapies. In addition, such cells could provide insights into human RGC development, gene regulation, and neuronal biology. Here, we report a simple, adherent cell culture protocol for differentiation of hPSCs to RGCs using a CRISPR-engineered RGC fluorescent reporter stem cell line. Fluorescence-activated cell sorting of the differentiated cultures yields a highly purified population of cells that express a range of RGC-enriched markers and exhibit morphological and physiological properties typical of RGCs. Additionally, we demonstrate that aligned nanofiber matrices can be used to guide the axonal outgrowth of hPSC-derived RGCs for *in vitro* optic nerve-like modeling. Lastly, using this protocol we identified forskolin as a potent promoter of RGC differentiation.

Diseases of the optic nerve often lead to progressive and irreversible vision loss. Glaucoma, the most common of the optic neuropathies, is the second leading cause of vision loss and blindness worldwide^1^,^2^. All current treatments for glaucoma are based on pharmacological, laser-based, or surgical approaches for lowering the eye’s intraocular pressure (IOP). Although such approaches can be effective, sufficient lowering of IOP is not always possible, and RGC loss can still progress despite lowered IOP. In order to develop improved treatment strategies for optic nerve disease, efforts are being made to better understand the mechanisms of axonal injury and RGC death, and to develop neuroprotective approaches to promote RGC survival^3^.

Many studies of RGC biology and disease mechanisms have utilized rodent model systems, either *in vivo* animal studies or *in vitro* studies of primary cultures of purified mouse or rat RGCs. Although such studies have provided many important insights, rodent RGCs have potential limitations for the understanding and treatment of human disease. Recent developments in the differentiation of human pluripotent stem cells (hPSCs) into retinal neurons allow for the investigation of human retinal disease using human cells as the model system^4^. Additionally, these advances may lead to development of cell-based therapeutic approaches based on hPSC-derived retinal cells^2^. The greatest progress in such studies has been with hPSC-derived retinal pigment epithelium (RPE)^5^ and photoreceptor cells^6^. Stem cell-derived photoreceptor cells that respond to light have been reported^7^, and clinical trials that utilize stem cell-derived RPE cell transplantation as a means to treat age-related macular degeneration (AMD) and Stargardt’s retinal degeneration have begun^5^.

Progress in the differentiation of hPSCs into RGCs has not advanced as rapidly as that of RPE and photoreceptors. Although successful RGC generation has been reported, most published studies have shown expression of a relatively small number of RGC-associated genes and limited physiological characterization of the derived cells, and most importantly, these studies have not provided a method to obtain highly purified populations of human RGCs in large numbers^7^,^8^,^9^,^10^,^11^,^12^,^13^,^14^. Here, we describe a simple and scalable protocol for differentiation of human embryonic stem cells (hESCs) to RGCs and their subsequent isolation and characterization. Using a CRISPR-Cas9 based genome editing strategy, we inserted an mCherry fluorescent reporter into the endogenous *BRN3B* (*POU4F2*) open reading frame (ORF) to mark differentiated RGCs. This reporter allows for the use of fluorescence-activated cell sorting (FACS) for generating a purified population of RGCs. We have confirmed that the differentiated cells express multiple RGC markers, exhibit morphological characteristics of RGCs at both the light and electron microscopic levels, demonstrate axonal trafficking of mitochondria, fire multiple spontaneous and induced action potentials, and express functional glutamate receptors. The differentiated RGCs also generate long aligned axons in culture when guided by a nanofiber matrix. Moreover, we identified the small molecule forskolin as a positive regulator of stem cell differentiation to RGCs. The availability of well-characterized and highly purified human RGCs will provide a useful cell resource for studying human optic nerve biology and disease, a more medically relevant system for drug discovery efforts, and may help in the development of novel cell-based therapies for the optic neuropathies.

## Results

### Development of an optimized protocol for differentiation of RGCs using an engineered hESC reporter cell line

**Figure 1:**
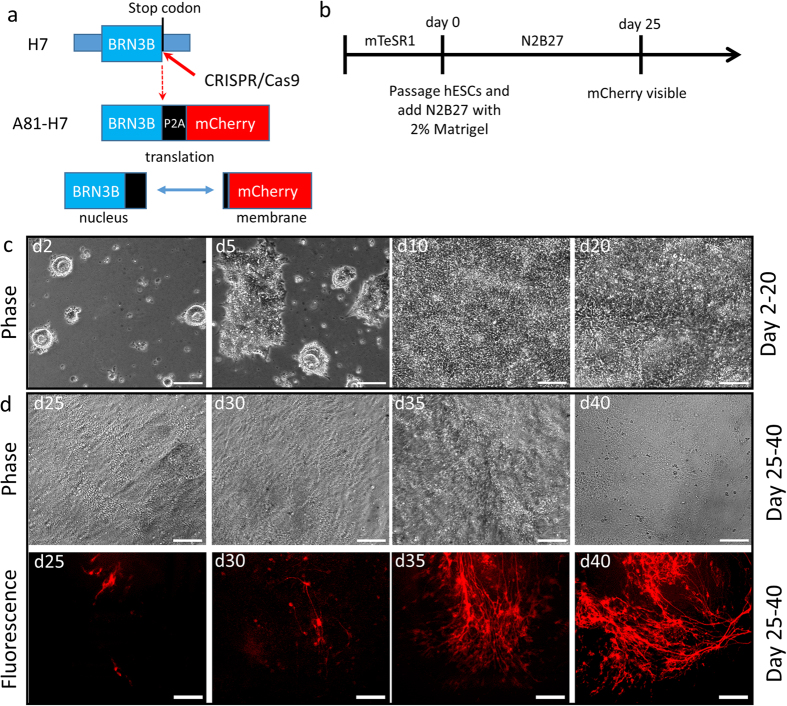
Generation and differentiation of an RGC reporter stem cell line. (**a**) Schematic illustration depicting the reporter design. CRISPR-Cas9 was used to target the stop codon of *BRN3B* in H7 hESCs. A P2A linked membrane targeted mCherry was added to the *BRN3B* ORF by homologous recombination. Following translation, the BRN3B transcription factor protein is localized to the nucleus while mCherry is targeted to the cell membrane. (**b**) Schematic of RGC differentiation protocol. (**c**) Phase microscopy of differentiation over time. Cultures become confluent by day 10. (**d**) Phase and fluorescence comparison microscopy of differentiation. mCherry fluorescence becomes visible on day 25. More mCherry+ cells form over time and cell bodies become clearly visible. **(c,d**) Scale bars = 100 μm.

With the goal of obtaining a population of purified human stem cell-derived RGCs, we generated a knock-in fluorescent mCherry reporter line in H7 hESCs using CRISPR-Cas9 genome editing^15^,^16^. We chose to modify the *BRN3B* gene locus for our reporter because BRN3B is an important and well-characterized transcription factor and RGC marker^17^,^18^ whose expression begins early in RGC differentiation and continues in adult cells. BRN3B is expressed in a large majority of RGCs, is RGC specific in the retina, and is relatively restricted in its expression throughout the rest of the body^17^,^18^,^19^. In order to maintain *BRN3B* expression and avoid creating a fusion protein of BRN3B-mCherry that could affect *BRN3B* function, we tethered together the *BRN3B* ORF and the mCherry fluorescent protein gene with a P2A self-cleaving peptide^20^. Additionally, we added a membrane signal peptide tag (GAP43 palmitoylation sequence) to the N-terminus of mCherry to guide this protein to the cell membrane. In this configuration, both proteins should be produced from one ORF while retaining their respective cellular localization and functional properties, and BRN3B should retain its normal expression levels. We designed a gRNA to target the stop codon of *BRN3B* and synthetized a template plasmid for recombination that contained 5′- and 3′- homology arms of the *BRN3B* locus and the P2A-mCherry target integration sequence (Fig. 1a). The expression plasmids for the gRNA, Cas9, and the BRN3B-P2A-mCherry template were electroporated together into H7 hESCs. The cells were then passaged using clonal propagation^21^, and seventy-two colonies were screened for reporter integration by PCR. One clone, named A81-H7, was found to be homozygous for the reporter (Fig. S1a) and free of predicted off-target mutations. This clone demonstrated a normal karyotype, as determined by G-band analysis of metaphase cells (Fig. S1b). All subsequent differentiation experiments were done using the A81-H7 cell line.

Rather than following one of the more established embryoid body-based methods of inducing retinal differentiation^8^,^9^,^10^,^12^,^13^,^22^,^23^, we modified the recently published adherent culture photoreceptor differentiation protocol of Boucherie *et al.* that relies on the default differentiation of hPSCs to the rostral neuronal phenotype in the presence of extracellular matrix (ECM) cues provided by the addition of Matrigel^24^. To simplify the protocol, we omitted factors that were included to promote photoreceptor differentiation: taurine, retinoic acid, sonic hedgehog (SHH), and fibroblast growth factors 1 and 2 (FGF1 and FGF2). We were particularly careful not to include factors that could potentially inhibit RGC differentiation^25^. To support our decision to eliminate these factors, we tested a subset of the factors and other supplements that could be beneficial, but observed no positive effect on RGC differentiation (Fig. S1c,d).

To initiate differentiation, A81-H7 hESCs were dissociated to small clumps of cells and plated on 1% Matrigel-coated dishes before being covered with N2B27 differentiation media containing 2% Matrigel (Fig. 1b). We observed formation of columnar neuroepithelial structures similar to those described in Boucherie, *et al.*^24^ (Fig. 1c, Movie S1). The cells filled out the entirety of the dish by day 10 and initial mCherry+ cells were evident by day 25 (Fig. 1d). The fluorescent signal labeled the entire cell body of the emerging cells, including the soma and neurites of the presumptive RGCs. At later time points, more mCherry+ cells appeared and these cells tended to cluster together and to send out fasciculated bundles of neurites throughout the dish (Fig. 1d, Fig. S2).

### Differentiating A81-H7 cultures mimic normal retinal development and express markers of all retinal lineages and cell types

**Figure 2:**
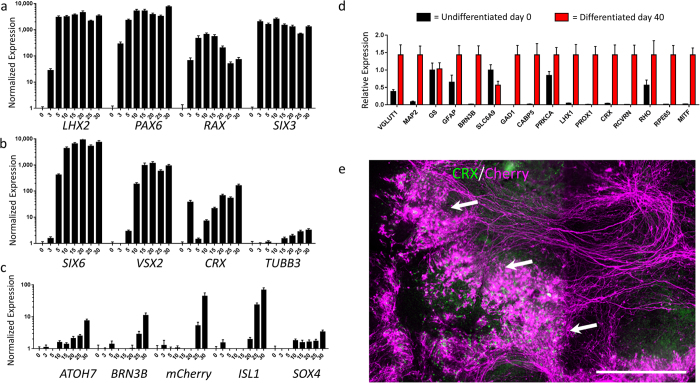
Retinal development is recapitulated in differentiating stem cells. (**a–c**) Temporal qPCR analysis of differentiation. Gene expression was first normalized to *GAPDH* and *CREBBP*, and then normalized to the value of undifferentiated A81-H7 hESCs. Error bars represent SEM. (**a**) Expression of eye field transcription factors. (**b**) Expression of optic vesicle, neural retina markers, and neuronal markers. (**c**) Expression of RGC-associated markers. (**d**) qPCR profile for presence of retinal cell types on day 40 of differentiation. Expression was normalized to *GAPDH* and *CREBBP*, A81-H7 d0 cells (black) were compared to differentiated cultures (red). Error bars represent SEM. Cell type markers: neurons – *VGLUT1*, *MAP2*; Müller glia-*GFAP*, *GS*; RGCs - *BRN3B*; amacrine cells - *GAD1, SLC6A9*; bipolar cells - *CABP5*, *PRKCA*; horizontal cells - *LHX1*, *PROX1*; photoreceptors - *CRX*, *RHO*, *RCVRN*; RPE - *MITF*, *RPE65*. (**e**) Immunostaining of day 49 cultures shows remnants of retinal organization. CRX+ photoreceptor progenitors in green and mCherry+ RGCs in magenta. CRX+ cells – white arrows - appear to segregate from mCherry+ axons, suggesting division between the outer nuclear layer and the nerve fiber layer. Scale bar = 500 μm.

To characterize the progression of retinal development of the A81-H7 cells during differentiation, we examined the expression of relevant retinal markers by quantitative real-time PCR (qPCR) over a 30-day time course. Upregulation of expression of 4 mammalian eye field transcription factors (*PAX6, LHX2, RAX, SIX3*)^26^ was observed by day 3 (Fig. 2a). By day 5, these genes had reached relative peak expression levels and their expression remained high over the course of differentiation, except for *RAX*, which decreased back down to day 3 levels by day 25, consistent with prior reports^27^. *SIX6*, a gene that comes on during optic vesicle formation^28^, was first detectable by day 5. This was closely followed by an increase on day 10 of expression of *VSX2* (Fig. 2b), a marker of neural retina formation^29^. There was also a spike in *CRX* mRNA expression on day 3 that was followed by a return to baseline on day 5; further *CRX* expression steadily increased over time (Fig. 2b). To test whether these early *CRX* transcripts resulted in detectable protein, we performed immunocytochemistry (ICC) for CRX on days 3 and 5 of differentiation, and observed the same pattern with high day 3 expression that decreased on day 5 (Fig. S3). This unusual biphasic pattern of expression may reflect the finding that there is early *CRX* expression in proliferating retinal cells, with *CRX* expression only becoming predominantly expressed in photoreceptors at later stages^30^. Lastly, we observed that post-mitotic neurons began to emerge starting on day 15, as indicated by an increase in expression of the *TUBB3* (βIII tubulin) gene. These results show that the eye field is formed early in this protocol, followed by optic vesicle-like differentiation, and then neural retina differentiation, suggesting that the cultures in general follow the pattern of normal retinal development.

Next, we explored the time course of expression of RGC-enriched genes (Fig. 2c). We quantified the expression of the transcription factors *ATOH7*, *BRN3B*, *SOX4*, and *ISL1*, as well as the mCherry reporter. One of the earlier genes to show a consistent increase in expression was *ATOH7*. This gene is expressed in progenitors of all retinal cell types^31^, but it is essential for the RGC cell fate as *ATOH7* knockout mice lack most of their RGCs and have diminished optic nerves^32^,^33^. Another gene that has recently been implicated in RGC genesis is *SOX4*^34^. *ATOH7* and *SOX4* both increased in expression starting on day 10, prior to the onset of *BRN3B* expression, which is consistent with previous reports that suggest that *ATOH7* and *SOX4* function upstream of *BRN3B*^32^,^34^. As expected, since they are transcribed as a common transcript, *BRN3B* and mCherry showed similar mRNA expression patterns, with expression being noted on day 25 and increasing into day 30. mCherry protein expression, as assessed by fluorescence, was also first detected on day 25. Lastly, we measured *ISL1* expression, whose encoded protein is known to interact with BRN3B to regulate RGC gene expression^35^. *ISL1* was strongly expressed by day 25.

A more comprehensive analysis of markers representing all major retinal cell types was performed with day 40 differentiated cells (Fig. 2d). Differentiated day 40 cultures showed a high level of *VGLUT1* and *MAP2* expression, suggesting a relatively mature neuronal phenotype. In addition to showing expression of RGC markers (*BRN3B*^36^), day 40 cells expressed markers for Müller glia (*GFAP*^37^), amacrine cells (*GAD1*^36^), bipolar cells (*CABP5*^36^, *PRKCA*^38^), horizontal cells (*LHX1*^36^, *PROX1*^7^), photoreceptor cells (*CRX*, *RHO*, *RCVRN*^24^), and RPE cells (*MITF*, *RPE65*^21^). Expression of glutamine synthetase (*GS*^37^) and solute carrier family 6 (*SLC6A9*^39^), markers of Müller glia and amacrine cells, respectively, was also detected, but without an increase over undifferentiated stem cells that already expressed these genes at a high level.

To explore whether the mCherry+ cells demonstrated spatial preferential localization with respect to other retinal cell types, we immunostained differentiated cultures for mCherry and CRX, a photoreceptor marker at later stages of differentiation (Fig. 2e). We observed that the somas of mCherry+ cells were interspersed with CRX+ cells, which were largely absent among the outgrowing mCherry+ neurites. This pattern suggests that the cultures may retain some degree of retinal organization with regards to a ganglion cell layer (GCL) and an outer nuclear layer (ONL). However, the CRX+ cells did not form a continuous laminar ONL-like structure as seen in the *in vivo* retina, as well as in spontaneously differentiated optic cups^9^,^10^. Unlike the 3-dimensional optic cup differentiation method^9^,^10^, our adherent cell-based culture method does not restrict cell migration within the dish, likely preventing cells from retaining a well-organized laminar retinal structure and promoting neurite outgrowth of RGCs throughout the dish.

### hESC-derived RGCs can be purified by FACS, survive long term, develop neurite networks in culture, and display ultrastructural properties of *in vivo* RGCs

**Figure 3:**
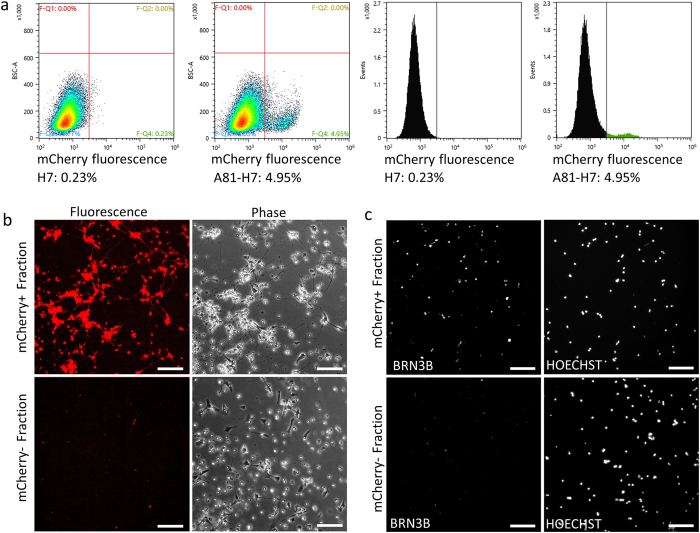
FACS purification of mCherry+ cells for culture and analysis. (**a)** FACS setup. Differentiated non-reporter H7 hESCs were used to set a threshold for mCherry fluorescence from A81-H7 cells. Density plots and histograms are displayed, with the percent of mCherry fluorescent cells indicated below. **(b)** Microscopy images of mCherry+ sorted cells one-day post FACS. Phase and fluorescence images are shown. Scale bar = 100 μm. **(c)** Fluorescence microscopy images of mCherry+ and mCherry- sorted cells. Cells were fixed and stained for BRN3B one day after sorting. Scale bar = 100 μm.

In order to obtain a purified population of RGC-like cells, we developed a protocol for flow-sorting the mCherry+ cells in a way that would maintain cell viability. Day 35 or older differentiated A81-H7 cultures were enzymatically dissociated to single cells. In order to develop appropriate FACS gates to define mCherry positive cells, we compared these cells to identically differentiated non-reporter H7 hESCs. We observed a contiguous and wide-spectrum of mCherry expression, with low levels of mCherry expression overlapping with the low level of auto-florescence observed from the non-reporter H7 cells (Fig. 3a). Using a gate that excluded greater than 99.5% of the non-reporter cells, 3–5% of the starting A81-H7 differentiated cell population was mCherry+. Repeat flow analysis of sorted cells right after sorting showed that the sorted population was highly enriched for mCherry+ cells, with 77.7% of cells registering above the threshold gate and an additional 20% registering on the edge of fluorescence (Fig. S4a). Examination of the sorted cells by fluorescence microscopy the day after sorting indicated that the mCherry+ population contained more than 90% fluorescent cells while the mCherry- population contained less than 0.5% fluorescent cells (Fig. 3b). Additionally, ICC performed on the mCherry+ cells with an anti-BRN3B antibody showed that approximately 90% of the cells were BRN3B+ (Fig. 3c). We also found that by increasing the fluorescence intensity level of the gate used for sorting we could increase the percentage of cells above the original fluorescence threshold to 95.6% (Fig. S4b). Taken together, these results demonstrate that FACS can be used to obtain highly purified populations of mCherry/BRN3B positive cells, and that the FACS parameters can be adjusted to achieve the desired balance between purity and yield.

**Figure 4:**
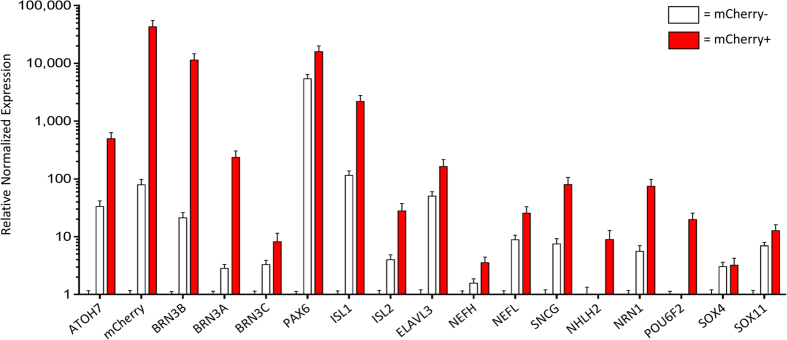
qPCR analysis of sorted cells. Day 40 sorted mCherry+/– cells were analyzed for RGC-associated genes. mCherry+ fraction shown by red bars, mCherry- fraction shown by clear bars. Expression was normalized to *GAPDH* and *CREBBP*, and then normalized to undifferentiated A81-H7 hESCs. Error bars represent SEM.

The sorted mCherry+ cells developed neurites within hours post-plating and the cells survived for months. The mCherry+ cells showed dynamic neurite extension and retraction over time (Movie S2, S3). In order to better image developing neurite morphology, we transduced the sorted cells with a constitutive expression GFP lentivirus to better highlight the dense neurite networks that developed between the cultured cells (Fig. S5a). The neurites developed in a pattern similar to what we have observed previously with primary rodent RGCs. We kept sorted cells alive for over a month after isolation and measured soma sizes on day 95 of culture (Fig. S6). The cells displayed large cell bodies (soma size: 18.1–26.0 μm, average: 21.2 μm, standard deviation: 2.6 μm, n = 20) which were very similar to human RGC sizes reported *in vivo*^40^. To establish whether the purified mCherry+ cells expressed markers characteristic of native RGCs, mCherry+ and mCherry- cell populations from sorted 40-day differentiated cultures were analyzed by qPCR. Expression data was normalized relative to A81-H7 undifferentiated stem cells (Fig. 4). In the mCherry+ cells, there was significant enrichment of expression of all three members of the *BRN3* transcription factor family^18^ as well as *ATOH7*, *PAX6*, and mCherry. Importantly, mCherry+ cells expressed *BRN3B* at more than 11,000- and 500-fold over the undifferentiated stem cells and mCherry- cells, respectively, further supporting the fidelity of the P2A reporter strategy. A number of other RGC markers also showed enrichment in the mCherry+ cells, including *ISL1*, *ISL2*, *ELAVL3* (also known as *HUC*), *NEFH*, *NEFL*, *SNCG*, *NHLH2*, *NRN1*, and *POU6F2*. As expected, the enrichment was highest for genes that are more specific to RGCs, such as *NRN1*, *POU6F2*, and *NHLH2*^41^,^42^,^43^, compared to *ELAVL3* and *NEFH/L*^44^,^45^. Lastly, since *SOX4* and *SOX11* knockout mice exhibit RGC loss, we looked for differences in expression of these genes in the sorted cells as well. However, the mCherry+ cells exhibited no *SOX4* enrichment and very little *SOX11* enrichment. Notably, these factors are also expressed in other retinal cell types^34^,^46^ and may only be RGC specific very early during differentiation. An analogous situation may explain our observations for the RGC markers *THY1* and *RBPMS*^47^,^48^,^49^. As expected, we observed strong expression of *THY1* and *RBPMS* in the mCherry+ cells (Fig. S5b). However, there was also high expression of these markers in the starting A81-H7 cell population and in the mCherry- cell population, consistent with the finding that expression of these genes outside the eye is not RGC-specific^49^,^50^,^51^. We also examined expression of *OPN4*, a marker of intrinsically photosensitive RGCs (ipRGCs)^52^. qPCR revealed a low but clearly detectable level of expression of *OPN4* (Fig. S5c) in the mCherry+ cells. A low level of expression in a mixed RGC population would be expected since ipRGCs represent only 1–2% of endogenous RGCs^52^.

**Figure 5:**
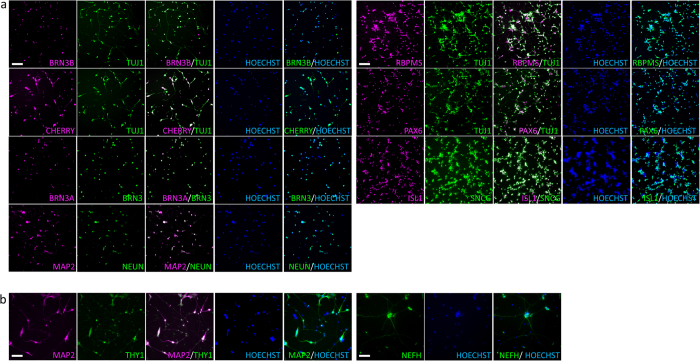
Sorted mCherry+ cells stain for RGC-enriched proteins. Fluorescence microscopy images of mCherry+ sorted cells. Cells were fixed and stained for the indicated RGC-enriched proteins one day after sorting **(a)** or 6 days after sorting (**b**). mCherry+ cells stained positive for the mCherry protein, the general neuronal markers TUJ1, MAP2, and NEUN, and the more RGC-enriched markers BRN3B, BRN3A, NEFH, PAX6, THY1, RBPMS, ISL1, and SNCG. The cells also stained with the pan-BRN3 antibody that recognizes all three BRN3 members. Hoechst was used for DNA staining. Scale bars = 100 μm.

We also tested the mCherry+ sorted cells for a variety of RGC-markers at the protein level (Fig. 5). ICC showed evidence of expression of the following RGC-associated proteins: pan-BRN3, BRN3B, BRN3A, NEFH, PAX6, THY1, RBPMS, ISL1, and SNCG, as well as the mCherry protein. The more general neuronal markers TUJ1, MAP2, and NEUN, which are expressed in RGCs, were also detected.

Additionally, we tested relative expression of non-RGC markers in the mCherry+ and mCherry- cell populations. In the mCherry- population we detected enrichment of non-RGC retinal markers for amacrine cells, bipolar cells, horizontal cells, and RPE (Fig. S7). To test for photoreceptor and Müller glia markers we immunostained the cells for CRX and GFAP, respectively, and did not observe expression of either marker in mCherry+ cells (Fig. S8).

**Figure 6:**
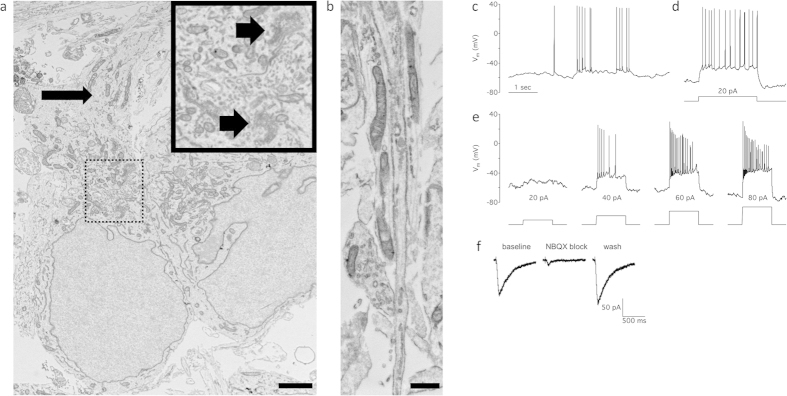
Sorted mCherry+ cells show ultrastructural and electrophysiological properties consistent with RGCs. (**a**) TEM of mCherry+ RGCs one week post-sorting. A81-H7 RGCs feature a large euchromatic nucleus, prominent rough endoplasmic reticula (arrowheads, bolded inset is a magnification of the dashed box), and an axon hillock (arrow). (**b**) TEM of neuronal processes showed that processes ranged in caliber from 0.2 to 2 μm. This representative micrograph displays processes containing longitudinally arranged neurofilament, mitochondria, and smooth endoplasmic reticula. Scale bars are (**a**) 2 μm and (**b**) 500 nm. (**c**) Spontaneous action potentials recorded in a cultured RGC under whole-cell current clamp. Time scale applies to all panels. (**d**) Injection of depolarizing current (bottom) causes the cell to fire a train of action potentials throughout the current injection (top), same cell as in (**c**). (**e**) A second representative example cell showing 20, 40, 60, or 80 pA depolarizing current injections (bottom: left to right) and the corresponding increases in the number of action potentials generated (top). V_m_ is membrane voltage. (**f**) Responses to 50 ms local dendritic puff applications of 100 μM kainate in a cultured RGC under whole-cell voltage-clamp (V_m_ = −70 mV). Left: average of baseline responses. Center: average of responses after bath application of AMPA/KA receptor antagonist NBQX (20 mM). Right: average of responses after NBQX washout.

We also assessed the FACS-sorted mCherry+ cells at the ultrastructural level by transmission electron microscopy (TEM). Consistent with observations of *in vivo* RGCs^40^, A81-H7 RGC somas demonstrated large euchromatic nuclei, prominent rough endoplasmic reticula, and axon hillocks (Fig. 6a). TEM of neuronal processes ranged in caliber diameter from 0.2 μm to 2 μm (Fig. 6b). While it was not possible to definitively differentiate axons from dendrites by TEM of adherent cultures of sorted RGCs, the neuronal processes contained elongated mitochondria, smooth endoplasmic reticula, and longitudinally-oriented neurofilaments, which are all features observed in RGC axons in the human nerve fiber layer and optic nerve^40^.

### FACS purified mCherry+ cells demonstrate physiological properties associated with mature RGCs

Calcium imaging was performed to assess the electrophysiological behavior of the sorted cells (Movie S4). Using the calcium sensitive dye Fluo-4AM, we observed spontaneous, transient spikes in intracellular calcium concentration in the sorted cells that appeared to be indicative of neuronal electrophysiological activity.

A hallmark of retinal ganglion cells, as compared to other retinal neurons, is their ability to encode membrane depolarizations in trains of action potentials. To determine whether the hESC-derived RGCs demonstrated this important feature, we performed whole-cell patch-clamp recordings. We observed that the RGCs were capable of spontaneous generation of action potentials driven by intrinsic membrane potential depolarizations (Fig. 6c). Furthermore, injecting current through the somatic recording electrode elicited action potentials in all cells studied (Fig. 6d). Nearly all (11/13) cells generated increasing numbers of action potentials in response to progressively larger current injections (Fig. 6e). The two remaining ganglion cells generated one or two action potentials. This distribution of cellular responses is consistent with previous studies in mammalian model systems examining RGC physiology at various stages of development^53^.

Ganglion cells receive excitatory, glutamatergic inputs from bipolar cells in the intact retina. To test whether cultured RGCs also express glutamate receptors, we utilized local pressure application (“puff”) of the AMPA/kainate receptor agonist kainate. A brief puff of kainate evoked a current that was reversibly blocked by the AMPA/kainate receptor antagonist NBQX (Fig. 6f). We confirmed this result in eight neurons from two separate cultures, confirming that these hESC-derived RGCs mature in culture such that they express the AMPA/kainate receptors necessary for excitatory transmission.

To test whether sorted RGCs display properties of axonal flow, we transfected the cells with an AAV virus mitochondria fluorescent reporter. Imaging of these cultures over time demonstrated mitochondria movement throughout RGC axonic processes (Movie S7).

### Aligned nanofiber scaffolds can guide RGC axonal development

**Figure 7:**
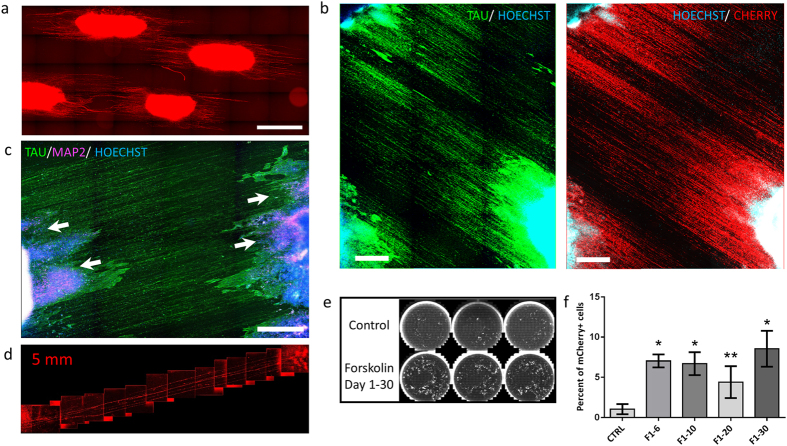
Differentiated cultures grow aligned axons on nanofiber scaffolds and addition of forskolin can improve stem cell differentiation to RGCs. (**a**) Fluorescence microscopy image for mCherry. Differentiated retinal cultures were dissociated and re-plated in Matrigel droplets on an aligned nanofiber scaffold that guides emerging neurite outgrowth. Scale bar = 1 mm. (**b,c**) Differentiated day 49 cultures were dissociated and plated on nanofibers in Matrigel droplets for 18 days before fixation. (**b**) Immunostaining for TAU and mCherry proteins. Hoechst staining shows that while cells were largely restricted in their migration, the cultures sprouted long projections that stained positive for TAU, suggesting axonal identity, and mCherry, suggesting RGC origin. Scale bar = 500 μm. (**c**) Immunostaining for TAU and MAP2 suggests that the RGC emanating projections are largely axonal since MAP2 appears to be restricted to the soma containing area of the dish (indicated by white arrows). Scale bar = 500 μm. (**d**) Matrigel droplets were loaded into a limited diffusion chamber of a 5 mm length. With limited diffusion, signaling cues from neighboring clusters may accelerate neurite outgrowth. (**e**) Whole-well microscopy scans of day 40 differentiated cultures. Addition of 25 μM forskolin from day 1 of differentiation until day 30 increases the number of differentiating mCherry+ cells as compared to control. (**f**) Flow cytometry analysis for percent of mCherry+ cells. Day 40 differentiated cells were treated with forskolin or DMSO for different times. Addition of 25 μM forskolin from day 1 of differentiation to day 6, 10, 20, or 30 increased the percentage of mCherry+ cells to similar extents, suggesting that the critical period for its mechanism of action is in the first week. *p < 0.01, **p < 0.05. N = 3. P values were 0.0005, 0.0032, 0.049, and 0.005, respectively. Unpaired two-tailed t-test was used to compare forskolin treated samples with control. Error bars represent standard deviation.

As a step toward modeling the optic nerve *in vitro*, we developed a system to guide RGC axonal outgrowth. Although hESC-derived RGCs demonstrate relatively long neurite-like processes in culture (Fig. S2), these processes generally appear disorganized, presumably due to the lack of appropriate extracellular matrix and target-derived axonal guidance cues. With these randomly arranged axon bundles, it would be difficult to measure axonal extension, injury response, and myelination behavior of the axons. Since aligned nanofiber scaffolds have been shown to effectively guide axonal outgrowth *in vitro*^54^, we tested whether RGC neurites could be guided to form long axons on polymer nanofibers that would better resemble the highly aligned nature of the endogenous optic nerve. In this study, we chose poly-ε-caprolactone (PCL) to prepare nanofibers due to its strong biocompatibility track record and the ease of processing for electrospinning^55^,^56^. Aligned PCL fibers with an average diameter of 630 nm were selected based on a pilot experiment showing that fibers with diameters in the range of 500 nm to 1.5 μm mediated the most effective directional migration for neurons (data not shown). These aligned fibers were surface coated with Matrigel (100 μg/ml) to improve their adhesion property to RGCs. We dissociated differentiated retinal cultures using brief enzymatic treatment to generate cell clusters and then encapsulated these clusters in Matrigel droplets plated on aligned PCL nanofibers (Fig. 7a). Robust fluorescent neurite extension, originating from the cell clusters, was observed along the nanofibers, and this outgrowth could be monitored over time using live cell imaging. It is clear from the video microscopy that axons were sampling and extending on the tracks of the aligned fiber substrate (Movie S5). To determine whether the long projections we observed were axonal, we immunostained the cultures for mCherry, TAU, and MAP2. The long projections were positive for mCherry and TAU, indicating that they originated from BRN3B+/RGC cells in the clusters (Fig. 7b). We then applied TAU/MAP2 staining to determine whether these two proteins had localized separately in the culture, an indication of axonal versus dendritic compartmentalization^57^. Indeed, the long projections stained positive for TAU, but negative for MAP2, confirming that they were axons. In contrast, consistent with the pattern seen in differentiated neurons^58^, MAP2+ dendrites were localized closer to the Matrigel encapsulated cell somas (Fig. 7c).

We also designed a limited diffusion chamber to enhance axon outgrowth in the differentiated RGC culture and to move closer to the development of an *in vitro* optic nerve model (Fig. 7d, Fig. S9). By placing two Matrigel encapsulated cell clusters in two microwells connected by a microchannel on top of an aligned nanofiber sheet (Fig. S9), we observed robust axonal outgrowth traversing the entire length of the chamber (5 mm) in 10 days, indicating an average axonal growth rate of about 0.5 mm/day. Interestingly, although the axonal growth rate is not known for human primary RGCs, the rate of axonal growth in embryonic day 15 mice has been reported to be about 1 mm/day^59^, a rate similar to the one that we observed under these *in vitro* culture conditions with hESC-derived RGCs. These data demonstrate that these hESC-derived RGCs can extend their axons in an organized fashion when guided by an artificial matrix.

### Addition of forskolin can improve stem cell differentiation to RGCs

Although the ability to purify BRN3B+ cells through the use of the mCherry reporter makes the efficiency of differentiation to RGCs less of an issue since more cells could be sorted overall, it would still be desirable to improve the efficiency of RGC differentiation beyond the 3–5% that we obtain with our first protocol. We therefore screened a number of small molecules in an attempt to increase the percentage of cells that differentiate to RGCs. Most of the tested molecules were ineffective, toxic, or inhibitory to RGC differentiation (Fig. S1c,d). However, we found that forskolin, an activator of adenylate cyclase that is known to increase RGC neurite outgrowth^60^ and survival^61^,^62^ as well as to augment differentiation in other systems^63^,^64^, had a dramatic positive effect on differentiation when added starting on day 1 (Fig. 7e). The effect of forskolin on RGC lineage commitment and differentiation appears to be mediated early, as exposure to forskolin for only the first week of culture had an effect comparable in magnitude to that observed when it was present continuously from day 1 to 30 (Fig. 7f, Fig. S10a). Moreover, the addition of forskolin starting on day 5 or later had no positive effect on RGC differentiation (data not shown). Since differentiation efficiency in Figure 7f was unusually low in the control, we repeated forskolin experiments additional times to validate its positive effect, and observed consistent RGC differentiation promoting activity (Fig. S11). We performed a dose-response experiment to establish an optimal forskolin dose, but saw limited effect from increasing the concentration from 5 to 50 μM (Fig. S10b). We also assessed the effect of forskolin on RGC marker expression. As measured by qPCR, sorted mCherry+ cells differentiated with the forskolin treatment protocol demonstrated enrichment for *BRN3A*, *BRN3B*, *BRN3C*, *PAX6*, *ISL1*, *ISL2*, *NHLH2*, and *POU6F2* expression that was similar to enrichment observed in mCherry+ cells generated in the absence of forskolin (Fig. S10c). Additionally, we checked the neurochemical behavior of forskolin-treated sorted cells by calcium imaging, and observed calcium transients similar to those observed in cells differentiated without forskolin (Movie S6). Lastly, in attempt to gain insight into the mechanism by which forskolin promotes RGC differentiation, we assessed gene expression changes by qPCR (Fig. S12). Perhaps surprisingly, forskolin upregulated expression of the eye field transcription factors on day 5 of differentiation, but on day 10 forskolin decreased their expression and increased expression of *SIX6* and *ATOH7*.

## Discussion

We have developed a simple and scalable system for differentiation and purification of human RGCs from hPSCs using a genetically engineered RGC reporter hESC line. To engineer the RGC reporter line, we utilized CRISPR-Cas9 technology to knock in a P2A-mCherry sequence into the 3′ end of the *BRN3B* ORF, an RGC marker gene. Then to derive RGCs we modified a recently published protocol designed for generation of photoreceptors^24^. This protocol appealed to us as a starting point because it does not require embryoid body formation, neural rosette picking, or tedious suspension cultures^7^,^9^,^10^,^14^,^22^,^23^, and yet it delivers efficient retinal differentiation. Since the protocol is an adherent culture protocol, it can easily be performed in 96 well plates for efficient screening or in tissue culture flasks of expanding sizes to generate larger numbers of cells. To increase RGC yield, we removed many additives that are unnecessary for RGCs, and which may actually inhibit RGC differentiation. Using our modified protocol and the engineered cell line, we first observed fluorescent cells appearing by day 25 of differentiation, which coincided with the timing of detectable BRN3B and mCherry mRNA expression. We then went on to characterize the RGC differentiation process over time. Our qPCR data suggests that the cultures followed the normal retinal development paradigm as has been described in other species: markers of the prospective eye field increased in expression early in the differentiation process, followed by markers of the “neural retina,” and finally markers of differentiated RGCs. Furthermore, on day 40 of differentiation, we detected markers of all retinal cell types by qPCR. In addition to retinal gene expression, we also found that the mCherry+ cells were localized in proximity to CRX+ cells in our cultures, suggesting that not only were the mCherry+/BRN3B+ cells likely to be of retinal origin themselves, i.e. RGCs, but also suggesting that the differentiating retinas had retained some measure of retinal organization despite the cultures having no rigid structure.

Building on these promising results, we developed a protocol to FACS purify the fluorescent cells. The sorted cells were characterized by qPCR and ICC for a wide range of RGC-enriched genes to confirm their cell identity. We found the sorted mCherry+ cells to be consistent with the RGC phenotype as they expressed all of the RGC markers we analyzed. However, few RGC markers, if any, are completely restricted to RGCs when compared to the nervous system and the rest of the body as a whole. For instance, although the *BRN3* transcription factors are restricted to RGCs in the retina, they are also expressed in the auditory system and the somatosensory system^19^. We confirmed that the sorted cells expressed *BRN3C* in addition to *BRN3B*. These two factors do not overlap in their spatial expression in the auditory system^19^, thus indicating that the sorted cells were not of auditory origin. Then to distinguish somatosensory neurons from our sorted cells, we profiled the mCherry+ cells for PAX6 by ICC and qPCR. *PAX6* is required for the development of the eye and the brain. It is expressed in all retinal progenitor cells but is restricted to RGCs and amacrine cells later in development^65^. While somatosensory neurons are not known to express *PAX6*, according to the BioGPS database^66^,^67^,^68^, our cells expressed PAX6 at a high level. Additionally, we noticed that our cells expressed *ISL1* more than *ISL2*, a pattern that also appears in retinal cells but not in the somatosensory system^66^,^67^. Moreover, the mCherry+ cells expressed *RBPMS*, a gene that has recently been suggested to be RGC-specific in the nervous system^48^,^49^. Based upon these results, we conclude that our mCherry+ cells are, indeed, of the RGC cell fate. Importantly, we also detected low-level expression of melanopsin (OPN4), a pigment protein expressed in a small subset of RGCs, i.e. ipRGCs^52^. It will be interesting to assess what other subtypes of RGCs can be detected in these cultures^69^, in order to establish whether certain subtypes are preferentially produced and if these preferences can be altered with small molecules or by transcriptional modulation.

In addition to expressing markers consistent with the RGC phenotype, mCherry+ cells also demonstrated ultrastructural properties consistent with *in vivo* RGCs as assessed by TEM, their mitochondria exhibited flow throughout the cells’ axons, and the cells appeared to be functional at the electrophysiological level. Using calcium imaging, we were able to detect calcium transients typical of neuronal electrical activity. Then, using whole-cell patch-clamp, we showed that the sorted cells fired multiple action potentials spontaneously and when exposed to current. Moreover, the cells demonstrated glutamate receptors as indicated by their ability to respond to the AMPA receptor agonist kainate, as expected for native RGCs. Together, this data suggests that these cells have the means to receive input and transmit output.

In an effort to build an *in vitro* optic nerve-like model and to provide a means to study RGC axonal outgrowth, we integrated our stem cell cultures with biomaterials to achieve axonal guidance. When differentiated retinal cultures were seeded onto nanofiber scaffolds, the cells responded to these scaffolds with guided axons growing for several millimeters in length in a time span of just two weeks. Due to their aligned nature, such scaffolds can be used to further probe RGC axonal guidance and through live imaging of the mCherry+ cells, axonal growth rates can be monitored in real time. Furthermore, this model may be used to better assess axonal repair following axonal injury in culture. It should therefore be possible to develop culture-based models of optic nerve injury and regeneration to partially mimic human disease. If RGCs are to be used for cell-based therapy in the future, engineered nanofibers may also provide a viable technology to guide the RGC axons to their proper targets in the brain. Additionally, it may be possible to enhance the activity of biomaterial scaffolds through conjugation of guidance molecules directly to the scaffolds, thereby combining physical and chemical guidance to increase and direct axonal growth^70^.

One of the advantages of incorporating a reporter into human stem cell-based differentiation paradigms is the ability to use reporter fluorescence as a readout in screens for differentiation modulating compounds. Through a pilot small molecule screen, we found that forskolin, when added early in differentiation, results in an increased percentage of RGCs. Forskolin is an activator of adenylate cyclase, which increases intracellular concentrations of cAMP. This molecule has been previously reported to improve RGC survival and promote neurite outgrowth^61^,^62^, and to aid in the direct reprogramming of fibroblasts to iPSCs^63^ and cholinergic neurons^64^. To explore forskolin’s activity on differentiating hESCs, we examined its effect on expression of selected retinal transcription factors. Notably, while neurogenesis did not appear to be affected as based on *NCAM1* expression, forskolin did increase expression of the eye field transcription factors on day 5, suggesting a pro-retinal effect. However, in the presence of forskolin on day 10, these genes had lower expression while *SIX6* and *ATOH7* were increased, perhaps suggesting an effect on optic vesicle or RGC formation. While the mechanism of forskolin’s RGC differentiation promoting activity requires further study, its addition to the protocol increases RGC generation and makes possible the production of 2.5 million RGCs per 24 well tissue culture plate. This brings the concept of using sorted human RGCs for large-scale drug screening closer to a practical reality. As large numbers of cells would be required for high throughput drug screening for studies of RGC survival, and for potential toxicology screens of compounds prior to clinical trials, we have begun a larger screen to identify additional small molecules that promote RGC differentiation. Identification of such molecules would not only allow for increased RGC yield, but would also provide molecular probes that could help further define RGC differentiation and biology.

The use of CRISPR-based gene editing approaches to make reporter lines, such as the BRN3B reporter described here, can also be used for the introduction of human mutations associated with optic nerve disease. An obvious early application of such technology will be the generation of human RGCs from ESCs and/or iPSCs carrying the E50K optineurin mutation in combination with the BRN3B reporter. E50K glaucoma patients appear to be particularly sensitive to RGC injury and death^71^. Development of a human RGC cell culture system that mimics E50K biology *in vitro* would allow the direct comparison of pertinent mutant versus wild-type cells and thereby aid greatly in efforts to elucidate novel disease mechanisms and develop potential therapeutics.

## Methods

### Plasmid construction

The CRISPR guide RNA (gRNA) and donor template plasmids used for the BRN3B-P2A-mCherry reporter line generation were constructed as previously described^16^. To generate the mCherry-targeting vector, we first synthesized a gBlock (IDT) encoding the P2A sequence, a *GAP43* palmitoylation sequence, and mCherry. This full sequence was inserted into the pUC19 vector (NEB) by Gibson Assembly (NEB). The left and right *BRN3B* homology arms, (898 base pairs) and (1,120 base pairs) respectively, were amplified from human genomic DNA using the following primers:

left forward: CGCCGAGGCTCTGGCAGCCG

left reverse: AATGCCGGCGGAATATTTCATTCTTTTC

right forward: TAGAAGACTCTTGGCCTCTCCAGAG

right reverse: TGCATCGGTCATGCTTCCAACTGC

These homology arms were inserted into the targeting vector using Gibson Assembly and the final donor vector was verified by sequencing prior to genome targeting. The targeting gRNA sequence (GCCAAGAGTCTTCTAAATGCCGG) was cloned into a U6-driven gRNA expression vector (Addgene #41824) as described^72^.

### Reporter line generation

Gene editing of H7 hESCs (WiCell) was performed as previously described with slight modifications^16^. Electroporation was performed using the Neon Transfection System 10 μL Kit (Invitrogen) according to the manufacturer’s instructions. Briefly, semi-confluent H7 hESC cells were cultured in mTeSR1 media (Stemcell Technologies) containing 10 μM Rho Kinase inhibitor DDD00033325 (EMD Millipore) 24 hours before passaging. H7 colonies were digested with Accutase (Sigma) for 5 minutes to form a single cell suspension. The cell suspension was centrifuged, and following supernatant removal, the cell pellet was left on ice for 15 minutes before resuspension in R-buffer containing three separate plasmids encoding the gRNA, Cas9 (Addgene #41815)^72^, and the donor template. The following electroporation parameters were used: voltage, 1,400 V; interval, 30 ms; 1 pulse. After electroporation, the cell suspension was slowly transferred to mTeSR1 medium containing 10 μM DDD00033325 and incubated at room temperature for 20 minutes before plating onto Matrigel (BD Biosciences) coated dishes. Cells were cultured until they reached confluency suitable for passaging, at which time they were split as a single cell suspension and grown at a low density of 500 cells per well of a 6-well plate. Once colonies formed, 72 individual colonies were manually picked and screened for reporter integration by PCR using the following forward and reverse primers (5′-3′):

forward: GGAGAAGCTGGACCTGAAGAAAAACGTGGTG

reverse: CCTTGGTGAAATCTAAAATCTGAAGGGCAAACACC

These primers amplify the genomic region containing the integration site, therefore showing if one or both alleles contain the reporter sequence. A homozygous reporter integration cell line was identified. This line, named A81-H7, was further validated by DNA sequencing and its karyotype was analyzed using standard procedures at Cell Line Genetics. To test this line for CRISPR off-target effects, we sequenced the 3 most likely off-targets and 2 additional predicted exonic off-targets as assessed by the CCTop online tool (Supplementary Table 2)^73^. All predicted sites were free of mutations.

### Human ESC maintenance

H7 and A81-H7 cells were maintained by clonal propagation in mTeSR1 media (Stemcell Technologies) on growth factor-reduced Matrigel coated plates^21^ at 10% CO_2_/5% O_2_. hESC colonies were passaged by dissociation with Accutase (Sigma) or TrypLE Express (Life Technologies) for 5 minutes, followed by ten-fold dilution of the cell suspension with DMEM/F12 (Life Technologies). After centrifugation at 150 × g for 6 minutes, the cell pellet was resuspended in mTeSR1 media containing 5 μM blebbistatin (Sigma). The cells were cultured at a density of 15,000 cells per well of a 6 well tissue culture plate. Two days after passage, the medium was replaced with mTeSR1, which was then changed daily.

### Human ESC differentiation towards RGC lineage

hESCs differentiation was induced as described previously^24^. Briefly, hESCs were incubated with TrypLE Express (Life Technologies) for 1 minute. Using a cell scraper (Sarstedt), colonies were lifted off the plate, transferred into DMEM/F12, and centrifuged at 150 × g for 6 minutes. The cell pellet was gently dissociated to a clump suspension via gentle pipetting in N2B27 differentiation media [1:1 mix of DMEM/F12 and Neurobasal (Life Technologies) with 1 × GlutaMAX Supplement (Life Technologies), 1 × antibiotic-antimycotic (Invitrogen), 1% N2 Supplement (Life Technologies), and 2% B27 Supplement (Life Technologies)]. The cell clump suspension was then distributed to a 24 well tissue culture plate (Falcon) pre-coated with 1% Matrigel. One well of a 6-well hESC culture was used per 24 well differentiation plate. After one hour at 37 °C in 5% CO_2_/20% O_2_, enough time for cell clumps to attach, media was replaced with cold (4 °C) N2B27 containing 2% Matrigel. Media was then changed every other day using N2B27 without Matrigel. The day of cell dissociation and 2% Matrigel addition is referred to as day 0 of RGC differentiation. For some experiments, 25 μM forskolin (Stemcell Technologies) was included in the media from differentiation day 1 to day 30 (unless otherwise specified). Other compounds we tested starting on day 10 were 25 ng/mL FGF-8 (PeproTech), 50 ng/mL FGF-A (PeproTech), 1 mM Taurine (Sigma), or 10% fetal bovine serum (Life Technologies).

### Fluorescence-activated cell sorting

To set the sorting gates, we used identically differentiated reporter-less H7 hESCs to define the mCherry+ cell population. Although H7 control cells exhibited low auto-fluorescent signal within our target gated population, we chose not to change this gate stringency since it would decrease yield. Side scatter height versus width linear alignment filters were used during sorting to minimize cell aggregates.

To prepare cells for sorting, differentiated cultures were dissociated into a single cell suspension. Differentiated cultures (day 35–63) were washed with phosphate buffered saline (PBS, pH 7.4) and then incubated with TrypLE Express for 15 minutes at 37 °C. The TrypLE was replaced with Accumax (Sigma) and incubated an additional 45 minutes. Cells were then gently triturated using a P1000 pipette, and DMEM/F12 was added to the cell suspension and centrifuged at 150 × g for 6 minutes. The cell pellet was resuspended in Live Cell Imaging Solution (Life Technologies) and further triturated. The single cell suspension was then passed through a cell strainer (BD Falcon) prior to analysis and sorting with an SH-800 Cell Sorter (Sony). Following sorting, the resulting cell suspension was centrifuged at 150 × g for 6 minutes. The cells were then resuspended in growth medium N2B27 with 10 μM forskolin and 10 ng/mL CNTF (PeproTech) and plated on Matrigel pre-coated plates. To prevent microbial contamination, 2 μg/mL doxycycline (Sigma) and/or 1 μg/mL mitomycin-C (Sigma) were added for 1 to 2 days of culture post sorting.

### Electrophysiology

To prepare cells for electrophysiology, FACS sorted cells from day 50 of differentiation were seeded on Matrigel coated glass coverslips (Fisher Scientific) inserted into culture plates at a density of 50,000–100,000 cells/cm^2^. Cells were fed with N2B27 media containing 2 μg/mL doxycycline, 1 μg/mL mitomycin-C, 10 μM forskolin, and 10 ng/mL CNTF. After 24 hours, the media was replaced with N2B27 supplemented with forskolin, CNTF, and doxycycline but without mitomycin-C. At 72 hours after plating, cell media was replaced with cold (4 °C) N2B27 media containing 1% Matrigel in order to prevent cells from detaching from the glass coverslip. The next day, media was replaced with N2B27 containing 10 μM forskolin, 10 ng/mL CNTF, 50 ng/mL BDNF (PeproTech), 10 ng/mL GDNF (PeproTech), and 10 ng/mL NT4 (PeproTech) and cultured in this media for 19–21 days until electrophysiology recording.

On day of recording, small portions of the coverslip were transferred to a microscope for electrophysiology experiments. Cells were superfused with Ames medium equilibrated with 95% O_2_/5% CO_2_, warmed to ~35 °C. Patch electrodes had tip resistances of 4–6 MΩ when filled with internal patch solution containing (in mM): 100 potassium methanesulfonate, 5 KCl, 5 NaCl, 10 HEPES, 4 EGTA, 10 phosphocreatine (Na salt), 4 Mg-ATP, and 0.4 Na-GTP. Access resistance was low (~10–15 MΩ) and was not compensated. Current-clamp recordings (Axopatch 1D amplifier, Axon Instruments) were acquired via a 16-bit Analog-digital interface (Instrutech Corp.; 10 kHz sample rate) and a low-pass filter (5 kHz). Data acquisition and analysis was controlled with custom macros written in Igor Pro (Wavemetrics). Extracellular recordings were collected prior to establishing the whole-cell configuration. For whole-cell current-clamp recordings, individual trials lasted 3–6 seconds and included 1–3.5 second step current injections generated by the patch-clamp amplifier. Successive trials varied the injected current from 20–140 pA, in 20 pA increments, to depolarize the membrane potential and to test for action potential responses. Some cells had a tendency to spontaneously depolarize and generate trains of action potentials at rest. For these cells, a small constant current was applied to hyperpolarize the membrane potential near −60 mV, beneath its spike threshold, thereby providing a clear baseline for subsequent action potential responses.

For kainate responses, whole-cell voltage-clamp recording were used, approximately 10 individual trials were averaged for a cell or particular response condition. Kainate (100 μM; Tocris) was dissolved in HEPES-buffered Ames and loaded into patch electrodes connected to a Picospritzer (Parker Instrumentation). The Picospritzer was triggered by the acquisition software to deliver 50–100 ms puffs of kainate (0.5 bars pressure) to proximal dendrites of recorded cells.

### Lentivirus production and transduction of sorted cells

Lentivirus was produced using calcium phosphate transfection as described^74^. One day before transfection 5 × 10^6^ 293T cells (Clontech) were seeded onto a 10-cm cell culture plate. The following day cells were transfected with 15 μg MD2.G (Addgene #12259), 6 μg MDL/pRRE (Addgene #12251), 6 μg RSV/rev (Addgene #12253), and 15 μg of the GFP-transfer vector (Addgene #17451)^75^. At 24 hours post transfection, 10 mM sodium butyrate was added to the cells. Lentivirus was harvested and concentrated at 48 hours post transfection using Lenti-X Concentrator (Clontech). To transduce sorted RGCs, lentivirus with 8 μg/mL Polybrene (Sigma) was added to the cell culture media overnight at 5–10 multiplicity of infection. The next day, the cells were washed with PBS and cultured with N2B27 media.

### Adeno-associated virus production and transfection of sorted RGCs

AAV2:mitoEGFPmCherry^76^ was produced and purified by triple transfection and heparin column purification^77^. The AAV cis-plasmid was pENN.AAV.CB7.CI:mito-EGFP-mCherry-SV40pA, the AAV trans-plasmid was p5E18 (pAAV2/2, Penn Vector Core), and pAdDeltaF6 (Penn Vector Core). The protocol from McClure, *et al.*^77^ was followed, except only AAV2 and not AAV1 was used in the transfection. Fluorescence images were taken one week after transfection.

### Nanofiber scaffold preparation and Matrigel droplet culture

Aligned polycaprolactone (PCL) nanofibers were prepared by electrospinning according to a previously reported method^78^. Briefly, PCL (MW 80 KDa, Sigma) was dissolved in hexafluoroisopropanol (HFP, Sigma) to obtain a solution of 17.5 w/v%. This solution was spun at a flow rate of 0.35 mL/hour through a blunt-end 27-gauge needle over a 42-mm separation distance towards a grounded rotating wheel (1,000 rpm), to which 15-mm glass coverslips were attached at a distance of 18 cm from the center or the wheel. A positive charge of 7 kV was applied to the needle tip (Gamma High Voltage Research). The average diameter of the fibers collected under these conditions was 632 nm ± 140 nm, as determined by ImageJ analysis of several scanning electron microscopy (SEM) images. The nanofibers on coverslips were sterilized by soaking in 95% ethanol, and then washed three times with PBS. The nanofiber coverslips were then coated with Matrigel (100 μg/ml) at 37 °C overnight before cell seeding.

Differentiated day 40 or older cells were dissociated to clusters by treating with Accumax for 10 minutes combined with gentle trituration. The cell clusters were collected by centrifugation and resuspended in 100 μL of pure ice-cold Matrigel solution. This cell cluster suspension was distributed to dry Matrigel pre-coated plates with nanofiber matrices and incubated at 37 °C for 20 minutes to allow the Matrigel droplets to form a hydrogel. The wells were then filled with N2B27 media supplemented with 10 μM forskolin and 10 ng/mL CNTF and refreshed every other day.

A limited diffusion culture was established to localize growth factor release between two neighboring cell clusters seeded on aligned nanofibers assembled within a microchannel in order to enhance axon network formation between the two clusters (Fig. S5). To prepare the nanofiber coverslips with a limited diffusion channel, nanofibers on the coverslips were trimmed to 10 × 2 mm rectangular sheets with nanofibers aligned along the long axis of the sheet. Both ends of the sheet were immobilized with silicone glue. A custom-made polydimethylsiloxane (PDMS, Sylgard 184) migration chamber consisting of two 3-mm diameter microwells with a connecting channel with dimensions of 10 mm (length) × 2 mm (width) × 100 μm (height) was placed over the nanofiber sheet. 1% Matrigel solution diluted in PBS was pipetted into the microwells and allowed to adsorb for 1 hour, and then washed with PBS before use. Cell clusters were seeded into microwells and then cultured according to the same protocol as described above.

### Calcium Imaging

Calcium imaging was performed as described previously^79^. Briefly, cells were purified by FACS on day 45 and cultured for 6 days before being loaded with 3 μM Fluo-4AM (Life Technologies), in HEPES-buffered physiological salt solution (PSS) containing 0.04% Pluronic F127 (Life Technologies) for 60 minutes at room temperature^79^. Cells were washed with PSS 3–5 times and then imaged using the EVOS FL Auto Cell Imaging System (Life Technologies). Images were taken every 3 seconds. ImageJ was used to analyze images.

### Quantitative real-time PCR

Total RNA was extracted using the RNeasy Mini Kit (Qiagen) and treated with RNase-free DNase I (Qiagen) to remove genomic DNA contamination. For FACS purified cells, approximately 100,000 sorted cells were used for each RNA extraction sample. Extracted RNA was reverse transcribed using the High Capacity cDNA Reverse Transcription Kit (Applied Biosystems). qPCR was performed with the CFX384 real-time PCR instrument (Bio-Rad) using the following program: 95 °C, 60 °C, and 72 °C – 40 cycles. All assays included technical and biological triplicates in 10 μL reactions using the Sso Advanced Universal SYBR Green Supermix (Bio-Rad) and 400 nM oligonucleotide primers. Primers used were designed by Geneious R7 (Biomatters) or sequences were obtained from publications^21^,^22^,^80^ or the PrimerBank database^81^ (Supplementary Table 1). Sample gene expression was normalized to the geometric mean of the reference genes *GAPDH* and *CREBBP*^80^. Relative normalized expression and the standard error of the mean (SEM) were calculated using Bio-Rad’s integrated CFX manager software.

### Immunocytochemistry

Cells were fixed with 4% paraformaldehyde in 0.2 M Sorenson’s phosphate buffer (pH 7.4) for 10 minutes at room temperature, washed three times with PBS, permeabilized with 0.1% Triton X-100 in PBS for 15 minutes, blocked using 2% bovine serum albumin (BSA) or 2% horse/goat serum in PBS for 1 hour at room temperature, and then incubated with primary antibody overnight at 4 °C with 0.1% Triton X-100. Primary antibodies used were TUJ1 (mouse, 1:2,000, Covance, MMS-435P), CRX (mouse, 1:10,000, Novus Biologicals, H00001406-M02), mCherry (rabbit, 1:200, Abcam, ab167453), TAU (chicken, 1:50, Abcam, ab75714), MAP2 (rabbit, 1:200, Santa Cruz, sc-20172), PAX6 (rabbit, 1:500, Covance, PRB-278P), THY1 (mouse, 1:100, Stemcell Technologies, 60045FI), BRN3 (goat, 1:50, Santa Cruz, sc-6026), BRN3A (mouse, 1:50, Santa Cruz, sc-8429), BRN3B (rabbit, 1:2000, Abcam, ab56026), RBPMS (rabbit, 1:50, Santa Cruz, sc-133950), ISL1 (rabbit, 1:100, Abcam, ab20670), NEFH (rabbit, 1:200, Abcam, ab8135), SNCG (mouse, 1:1,000, Abnova, H00006623-M01), and NEUN (mouse, 1:50, Millipore, MAB377). Cells were incubated with species-specific corresponding secondary antibodies for 45 minutes at room temperature. Secondary antibodies used included Alexa Fluor-488 and 647 conjugated antibodies (1:1,000, Life Technologies). Hoechst 33342 (trihydrochloride, trihydrate, Life Technologies) was used to stain nuclei. Fluorescence images were acquired with the Eclipse TE-2000S inverted microscope (Nikon) and the EVOS FL Auto Cell Imaging System. Identical samples, without the primary antibody added, were used as controls for antibody specificity.

### Transmission electron microscopy (TEM)

FACS sorted cells from day 40 of differentiation were seeded on Matrigel coated glass coverslips in 35 mm tissue culture dishes at a density of 50,000–100,000 cells/cm^2^. Cells were fed with N2B27 media containing 2 μg/mL doxycycline, 1 μg/mL mitomycin-C, 10 μM forskolin, and 10 ng/mL CNTF. After 24-hours, the media was replaced with N2B27 supplemented with forskolin, CNTF only. Seven days post plating the cells were fixed for 1 hour in 2.5% glutaraldehyde/2% paraformaldehyde in 0.1 M sodium cacodylate buffer, pH 7.4 (all from Electron Microscopy Sciences) at room temperature and overnight at 4 °C. Cells were post-fixed with ferrocyanide (Sigma-Aldrich)-reduced osmium tetroxide (Electron Microscopy Sciences), stained with 1% thiocarbohydrazide (Polysciences, Inc.) followed by 2% osmium tetroxide. Cells were subsequently stained with 1% uranyl acetate (Electron Microscopy Sciences) and 0.66% lead nitrate (Electron Microscopy Sciences) in 0.03 M aspartic acid, pH 5.5 (Sigma-Aldrich) before progressive dehydration into ethanol. Durcupan (Sigma Aldrich) embedded cells were hardened for 36–48 hours at 60–65 °C. 60 nm-thick sections were imaged using an H7600 transmission electron microscope (Hitachi).

### Live cell imaging

The EVOS FL Auto Cell Imaging System was used for imaging cells in culture over time and for scanning whole live culture plates. During imaging experiments, cells were maintained in a live cell chamber at 37 °C with 5% CO_2_ and 85% humidity.

## Additional Information

**How to cite this article**: Sluch, V. M. *et al.* Differentiation of human ESCs to retinal ganglion cells using a CRISPR engineered reporter cell line. *Sci. Rep.* **5**, 16595; doi: 10.1038/srep16595 (2015).

## Supplementary Material

Supplementary Movie S1

Supplementary Movie S2

Supplementary Movie S3

Supplementary Movie S4

Supplementary Movie S5

Supplementary Movie S6

Supplementary Movie S7

Supplementary Information
