# Supplementary material for: Differentiation of human ESCs to retinal ganglion cells using a CRISPR engineered reporter cell line
Source: Sci Rep. 2015 Nov 13;5:16595. doi: 10.1038/srep16595 (PMC4643248; doi:10.1038/srep16595)
Supplement: Supplementary Information [file srep16595-s8.pdf]

Supplementary Information

Title: Differentiation of human ESCs to retinal ganglion cells using a CRISPR engineered reporter cell line

Authors: Valentin M. Sluch[1], Chung-ha O. Davis[2], Vinod Ranganathan[3], Justin M. Kerr[4], Kellin Krick[5,6], Russ Martin[6,7], Cynthia A. Berlinicke[3], Nicholas Marsh-Armstrong[2,8], Jeffrey S. Diamond[4], Hai-Quan Mao[5,6,7], Donald J. Zack[1,3,8,9]*

1. Department of Molecular Biology and Genetics, Johns Hopkins University School of Medicine Baltimore, MD 21287

2. Hugo W. Moser Research Institute, Kennedy Krieger Institute, Baltimore, MD, 21205

3. Department of Ophthalmology, Wilmer Eye Institute, Johns Hopkins University School of Medicine, Baltimore, MD 21287

4. Synaptic Physiology Section, National Institute of Neurological Disorders and Stroke, National Institutes of Health, Bethesda, MD 20892

5. Department of Biomedical Engineering, Johns Hopkins University School of Medicine, Baltimore, MD 21205

6. Translational Tissue Engineering Center, Johns Hopkins University School of Medicine, Baltimore, MD 21287

7. Department of Materials Science and Engineering, Whiting School of Engineering, and Institute for NanoBioTechnology, Johns Hopkins University, Baltimore, MD 21218

8. The Solomon H. Snyder Department of Neuroscience, Johns Hopkins University School of Medicine, Baltimore, MD, 21205

9. Institute of Genetic Medicine, Johns Hopkins University School of Medicine, Baltimore, MD 21287

Author for correspondence: Donald J. Zack, M.D., Ph.D., 400 N. Broadway, Smith Building, Room 3029, Baltimore, MD 21231. dzack@jhmi.edu. 410 502-5230

Figure S1. Genetic analysis of A81-H7 cells and effect of FBS, Taurine, FGF-8, and FGF-A on differentiation to mCherry+ RGCs. (a) PCR test for homozygosity. Primers spanning the integration region were used to amplify genomic DNA for comparison between the parental H7 line and the isolated A81-H7 clone. A81-H7 DNA produced only one band of expected integration size, indicating homozygosity of the modified locus. **(b)** G-banded karyotype analysis of A81-H7 cell line. **(c,d)** Whole-well microscopy images of day 37 differentiated cultures. **(c)** FGF-A 50ng/mL was added to differentiating cultures. **(d)** Fetal bovine serum (FBS) - 10%, Taurine – 1 mM, FGF8 – 25 ng/mL or a combination of all three treatments were added to differentiating cultures from day 10 to 30. No visible effect on RGC differentiation was detected from these treatments as compared to the negative control.

Figure S2. Differentiated mCherry+ culture. Stitched microscopy images on day 38 of differentiation. At this point, mCherry+ cells appear as dense clusters from which fasciculated bundles of neurites extend throughout the dish. Scale bar= 500 µm.

Figure S3. CRX protein expression early in differentiation. Immunofluorescence microscopy images of differentiating cultures on days 3 and 5. Cells were fixed and stained for CRX and Hoechst was used to stain nuclei. Scale bar= 100 µm.

Figure S4. Flow analysis of sorted cells. (a) Day 35 differentiated cells were purified by FACS and then analyzed by flow cytometry. Percent red is determined from a threshold set based on fluorescence of non-reporter H7 cells. Sorted cells are 77.7% above the initial sorting threshold (vertical line), and are 97.76% positive if cells on the edge of fluorescence are

included. **(b)** Cells were sorted for higher fluorescence as based on the gate shown in red, and then analyzed by flow cytometry with 95.58% of cells being above the non-reporter H7 fluorescence threshold.

Figure S5. FACS sorted cells develop neurite networks and express RGC markers. (a) Microscopy images of a long term culture of FACS-purified mCherry+ cells. Cells were transduced with lenti-GFP and cultured for 18 days. GFP fluorescent dense neurite networks are visible. Scale bar=100 µm. **(b,c)** qPCR analysis of sorted mCherry+ cells from day 40 differentiated cultures. Expression was normalized to *GAPDH* and *CREBBP*. Error bars represent SEM. mCherry+/- cells were compared to undifferentiated A81-H7 hESCs. **(b)** All three cell populations express the RGC-enriched markers of *THY1* and *RBPMS*. Although *THY1* and *RBPMS* are specifically expressed in RGCs within the retina, they are also expressed in a number of other tissues.[48-50] **(c)** Only mCherry+ cells express the ipRGC marker *OPN4* (melanopsin). Two different primer pairs were used to confirm *OPN4* expression.

Figure S6. FACS sorted cells survive long term and develop long neurites. (a,b) Microscopy images of a long-term culture of FACS-purified mCherry+ cells. Cells were transduced with lenti-GFP after sorting on day 43 of differentiation and cultured for 52 days post-sort. Scale bars are **(a)** 100 µm and **(b)** 500 µm.

Figure S7. qPCR analysis of sorted cells. Day 63 sorted mCherry+/- cells were analyzed for expression of retinal cell type associated genes. mCherry+ fraction shown by red bars,

mCherry- fraction shown by clear bars. Expression was normalized to *CREBBP*. Error bars represent SEM.

Figure S8. Immunostaining analysis of sorted cells. Sorted mCherry+ cells stain negative for CRX and GFAP while some mCherry- cells stain positive.

Figure S9. Schematic representation of limited diffusion chamber for RGC culture on aligned nanofibers. (a) Aligned nanofibers were electrospun onto coverslips and coated with matrigel. **(b)** The migration chamber was placed over the fiber sheet. **(c)** RGCs in matrigel solution were pipetted onto fibers in clusters at each end of the chamber. **(d-e)** Chamber was filled with media and cells were further cultured, allowing for axon extension along the fibers.

Figure S10. Effect of forskolin treatment on differentiated cultures. (a) Whole-well microscopy images of day 40 differentiated cultures used for flow cytometry analysis in **Fig. 6f**. Cultures were treated with 25 µM forskolin from day 1 to day 6, 10, 20, or 30 as compared to a DMSO control group. **(b)** Forskolin dose response. Differentiated cultures were treated with increasing doses of forskolin from 2.5 to 50 µM for days 1 to 6 as compared to DMSO. Increased mCherry+ cells were observed with with forskolin of 5 µM or higher, with no visibly consistent differences noted between 5-50 µM forskolin. **(c)** qPCR analysis of forskolin treated differentiated day 40 cells. Forskolin was applied from day 1 to 6 of differentiation. Gene expression was normalized to *GAPDH* and *CREBBP*. mCherry+ cells were normalized to mCherry- cells. Error bars represent SEM. mCherry+ cells show enrichment for expression of

the RGC-associated genes *BRN3A*, *BRN3B*, *BRN3C*, *ISL1*, *ISL2*, *NHLH2*, *POU6F2*, and *PAX6*.

Figure S11. Forskolin increases percent of RGCs in differentiation. Flow cytometry analysis for percent of mCherry+ cells in differentiated cultures at day 40 and day 35. Cells were treated with 25 µM forskolin or DMSO from days 1 to 6 of differentiation. **$p<0.05$. N=3. P values were 0.04 and 0.01, respectively. Unpaired two-tailed t-test was used to compare forskolin treated samples with control. Error bars represent standard deviation.

Figure S12. Analysis of forskolin induced gene expression changes during differentiation. Differentiating cultures were treated with either DMSO or 25 µM forskolin for 10 days and RNA was extracted and analyzed on days 5 and 10. Expression was normalized to *GAPDH* and *CREBBP*. Error bars represent SEM. CFX Manager software was used to perform a two-tailed t-test to calculate the p-values. **$p<0.05$, *$p<0.01$. N=3. P values for day 5 were: *SIX3* - 0.000056, *LHX2* - 0.010169, *PAX6* - 0.021039, *RAX* - 0.000021, and *ATOH7* - 0.002231. P values for day 10 were: *SIX3* - 0.000029, *LHX2* - 0.000007, *RAX* $p< 0.000000$, *SIX6* - 0.000195, *VSX2* - 0.013346, *ATOH7* - 0.032986, and *SOX4* - 0.001031.

Movies

Movie S1. Formation of neuroepithelium in response to Matrigel treatment. Following cell clump plating and administration of the Matrigel cover layer, the cell cultures were moved to the EVOS FL Auto Cell Imaging System stage and imaged for 88 hours using 20x magnification with a phase image taken every 15 minutes.

Movie S2. FACS sorted RGCs display dynamic neurite outgrowth one day post sorting. Cultures were differentiated for 40 days before sorting. An image was taken every 30 min for 39 hours at 20x magnification using a phase objective.

Movie S3. FACS sorted RGCs continue to display dynamic neurite outgrowth one week post sorting. Cultures were differentiated for 40 days before sorting and then cultured for 7 days before imaging using the EVOS system. An image was taken every 30 min for 24 hours at 20x magnification using a phase objective.

Movie S4. Calcium imaging of sorted cells. Differentiated cultures were sorted on day 45 and maintained for 6 days before addition of the calcium sensitive dye Fluo-4AM. Using the EVOS system, images were taken every 3 seconds using a 20x objective to observe calcium transients in absence of an exogenous stimulus.

Movie S5. Monitoring neurite outgrowth on aligned nanofibers using mCherry fluorescence. Differentiated cultures were dissociated and plated as clusters in Matrigel droplets onto nanofiber containing dishes. These cultures were maintained for 13 days before live imaging using the EVOS system. A fluorescence image was taken every 15 minutes for 24 hours using a 20x objective. ImageJ was used to invert the fluorescence images before stacking to create the movie.

Movie S6. Calcium imaging of cells differentiated in the presence of forskolin. Stem cell cultures were treated with forskolin from day 1 to 6 of differentiation and sorted on day 38. The sorted cells were maintained for an additional 7 days before calcium imaging. Images were taken every 3 seconds using a 10x objective to observe calcium transients in the absence of an exogenous stimulus.

Movie S7. FACS sorted RGCs display axonal flow of mitochondria. Sorted mCherry+ cells were transduced with AAV virus carrying a mitochondria red-green reporter. Red fluorescence images were taken one week after transfection with an image taken every 15 min for 18 hours using the EVOS system.

Figure S1

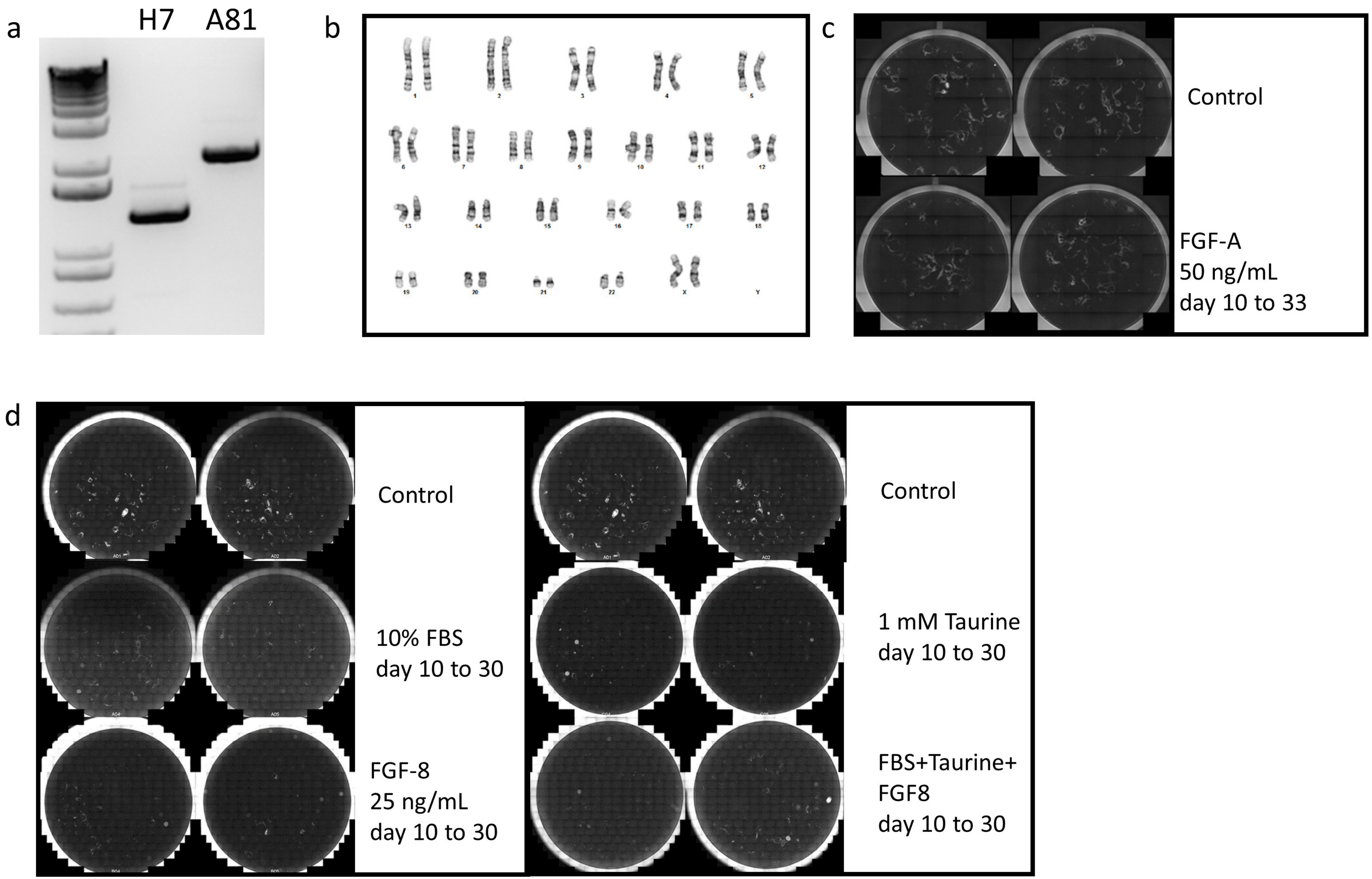
a
H7
A81
b
c
Control
FGF-A
50 ng/mL
day 10 to 33
d
Control
10% FBS
day 10 to 30
FGF-8
25 ng/mL
day 10 to 30
Control
1 mM Taurine
day 10 to 30
FBS+Taurine+
FGF8
day 10 to 30

Figure S2

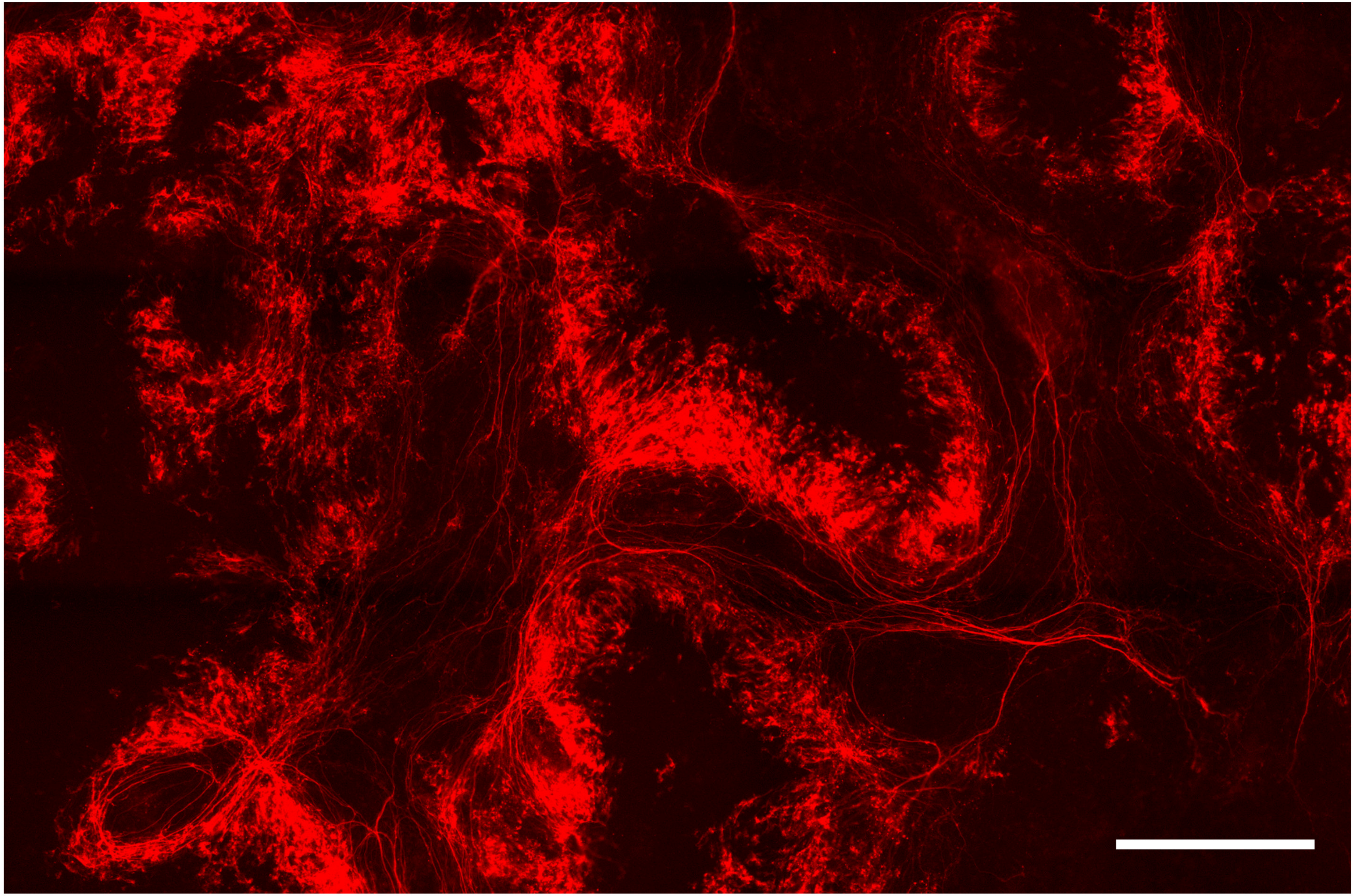

Figure S3

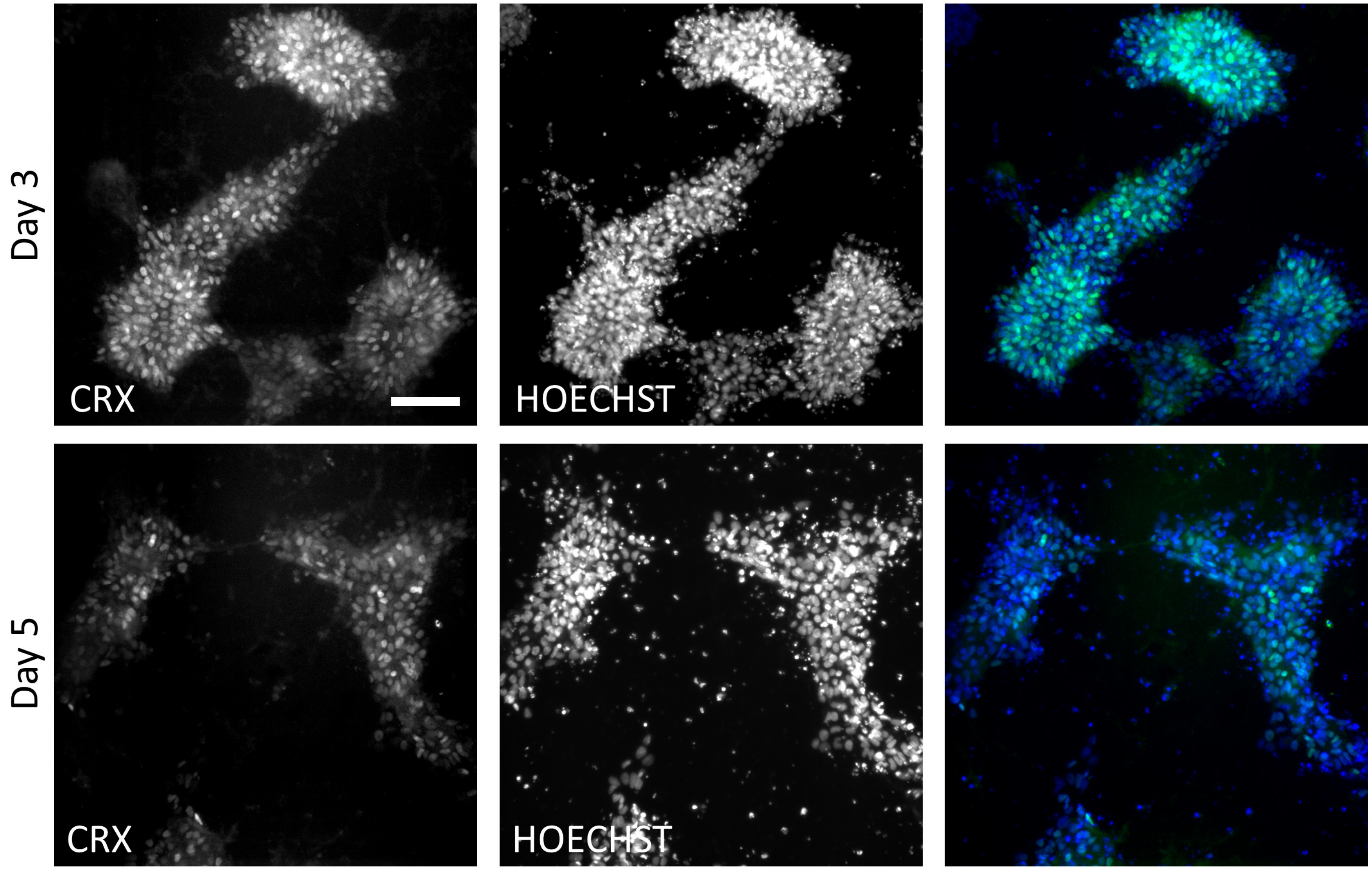

Figure S4

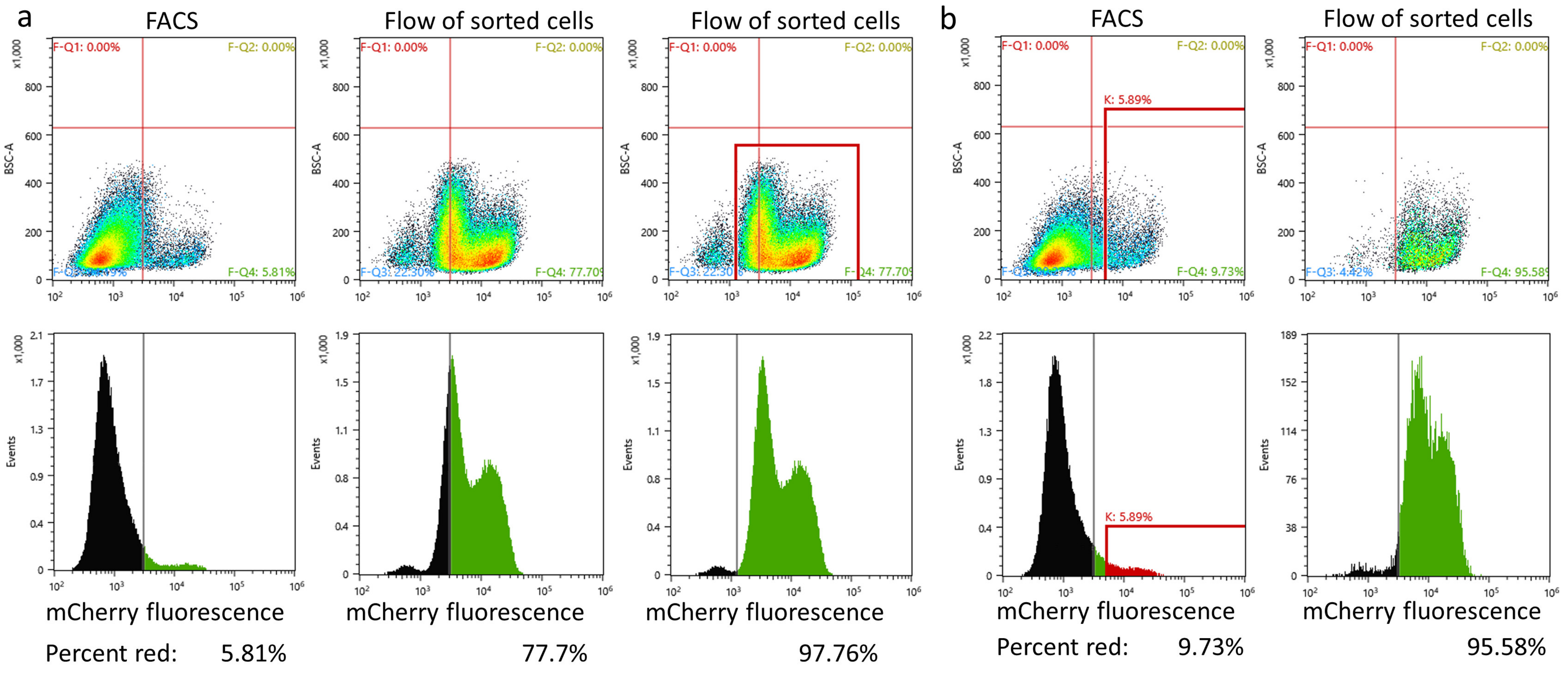
a
FACS
Flow of sorted cells
Flow of sorted cells
b
FACS
Flow of sorted cells
F-Q1: 0.00%
F-Q2: 0.00%
F-Q4: 5.81%
F-Q3: 22.30%
F-Q4: 77.70%
K: 5.89%
F-Q4: 9.73%
F-Q3: 4.42%
BSC-A
Events
x1,000
mCherry fluorescence
mCherry fluorescence
mCherry fluorescence
mCherry fluorescence
mCherry fluorescence
Percent red: 5.81%
77.7%
97.76%
Percent red: 9.73%
95.58%

Figure S5

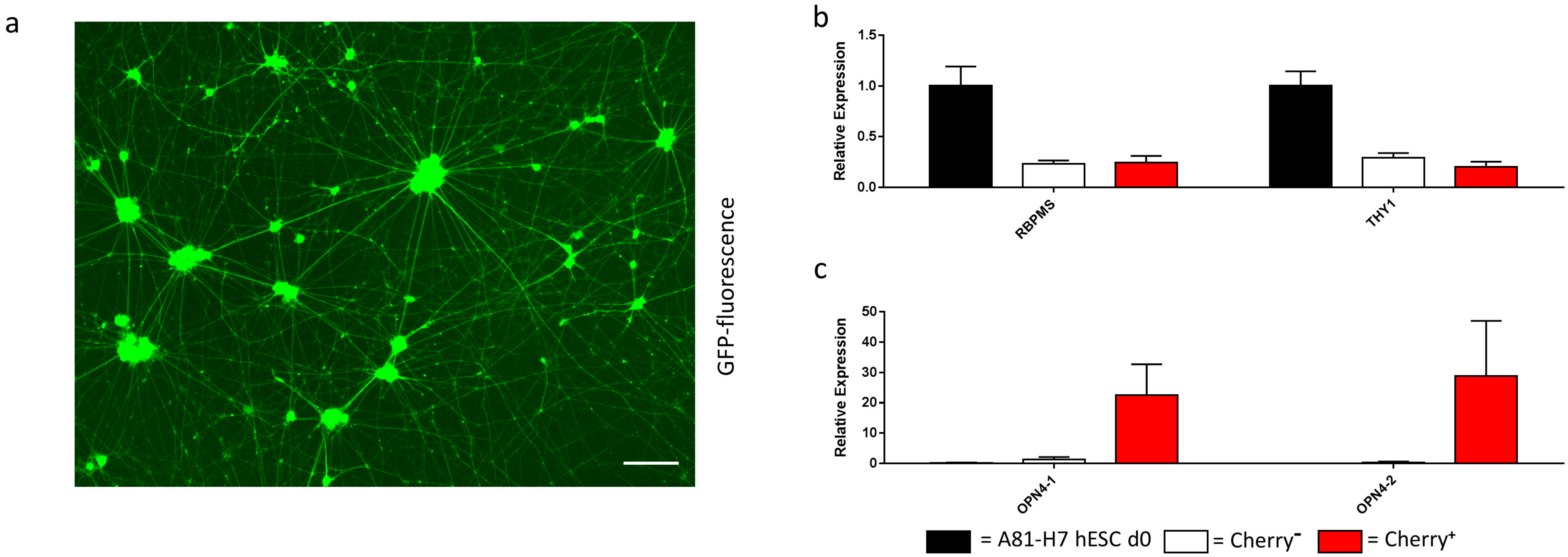
a
GFP-fluorescence
b
Relative Expression
1.5
1.0
0.5
0.0
RBPMS
THY1
c
Relative Expression
50
40
30
20
10
0
OPN4-1
OPN4-2
= A81-H7 hESC d0
= Cherry-
= Cherry+

Figure S6

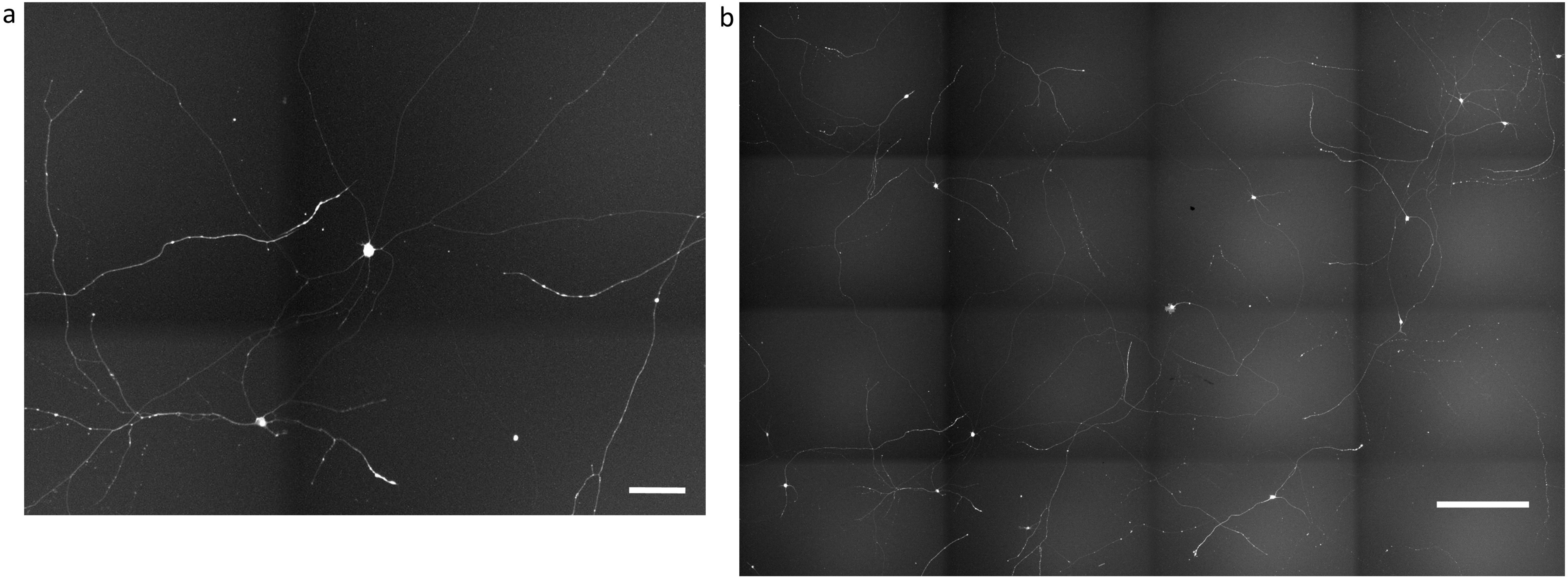

Figure S7

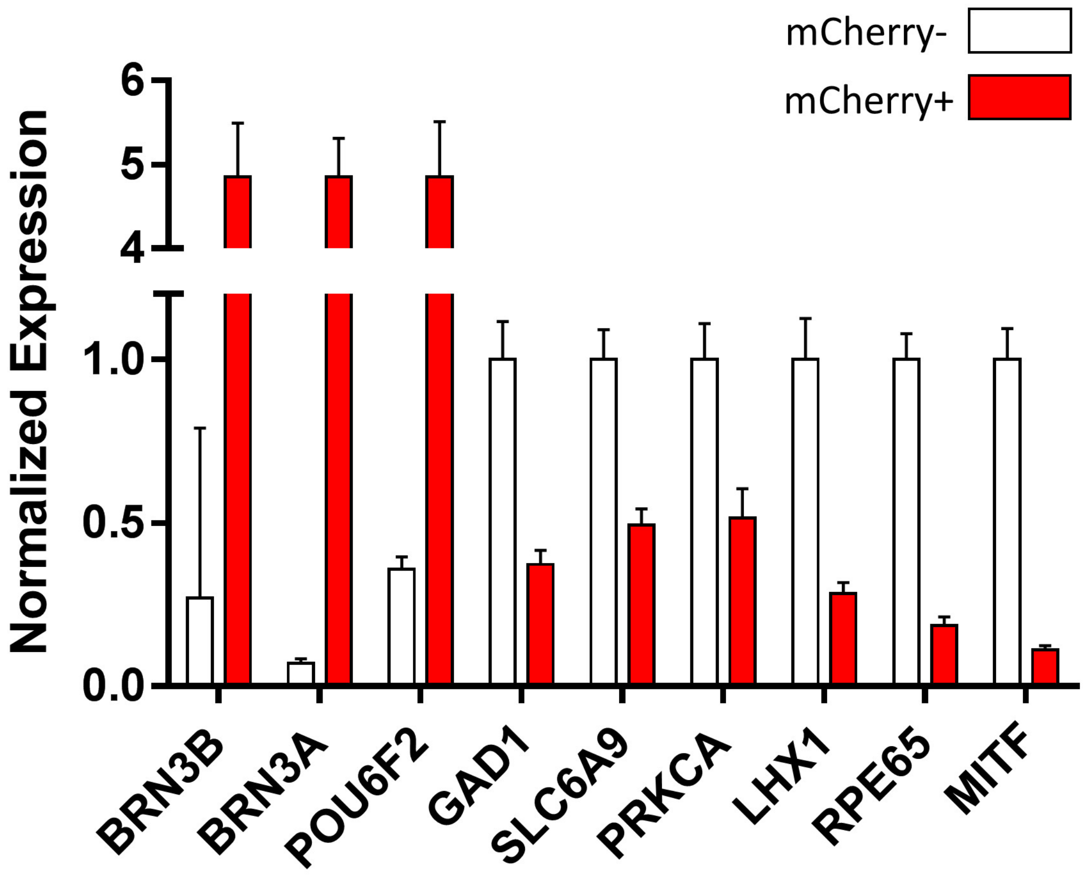
mCherry-
mCherry+
6
5
4
1.0
0.5
0.0
Normalized Expression
BRN3B
BRN3A
POU6F2
GAD1
SLC6A9
PRKCA
LHX1
RPE65
MITF

Figure S8

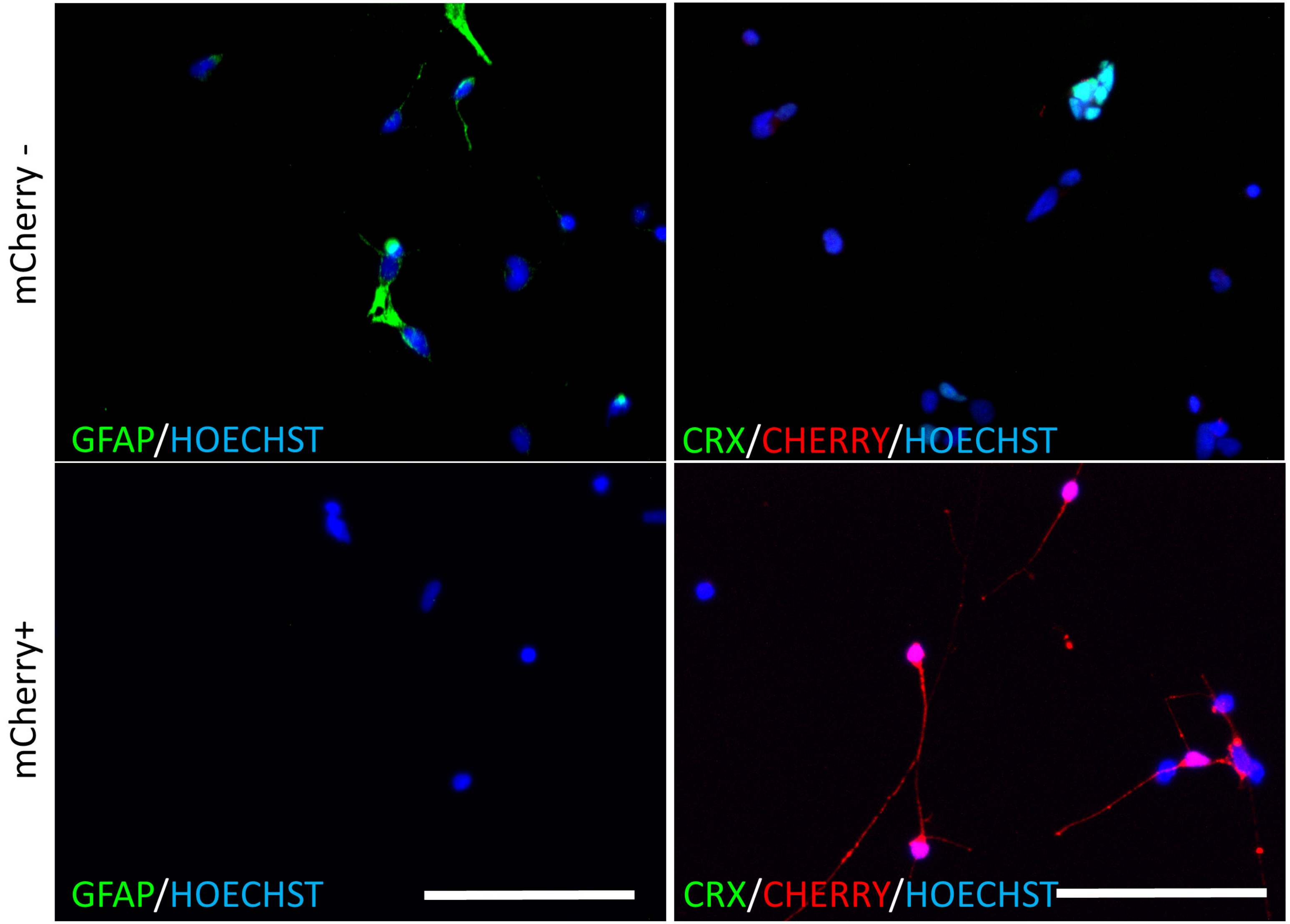

mCherry -
mCherry+
GFAP/HOECHST
CRX/CHERRY/HOECHST
GFAP/HOECHST
CRX/CHERRY/HOECHST

Figure S9

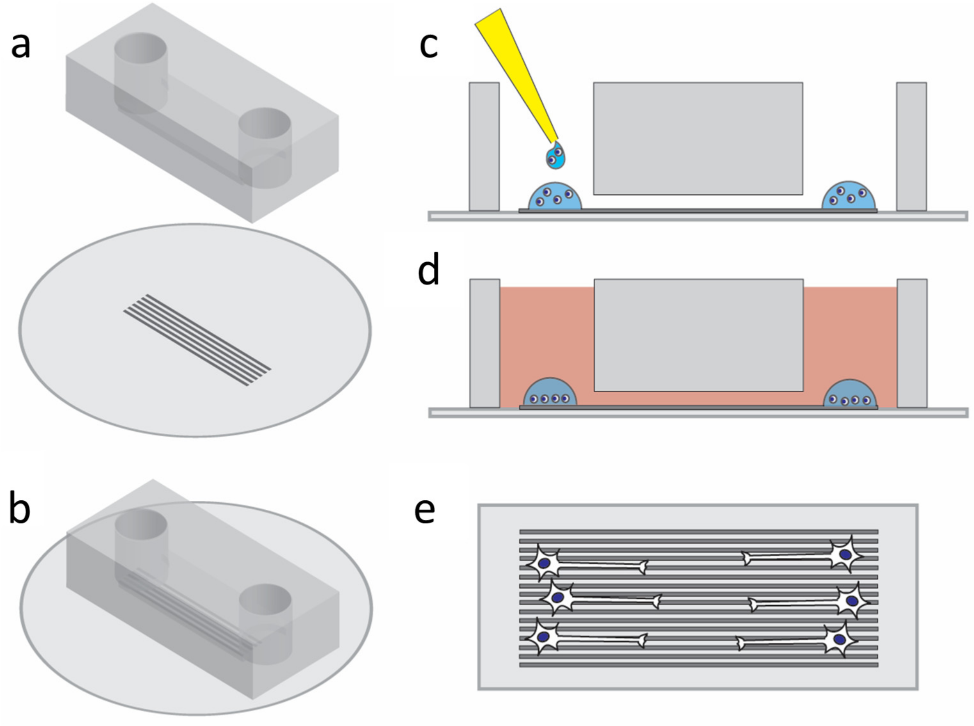

Figure S10

a

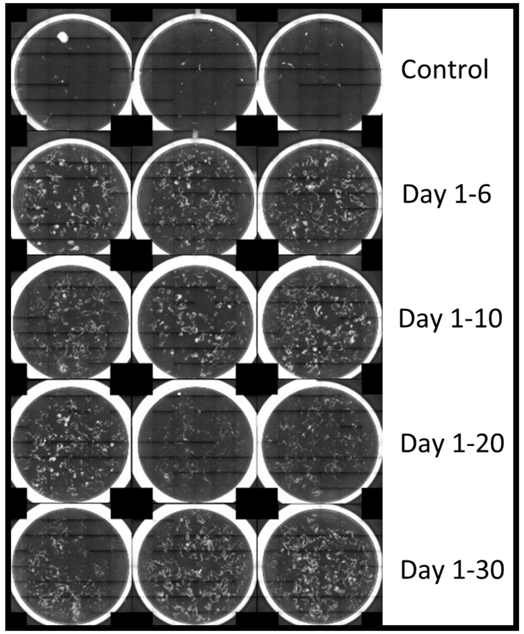

b

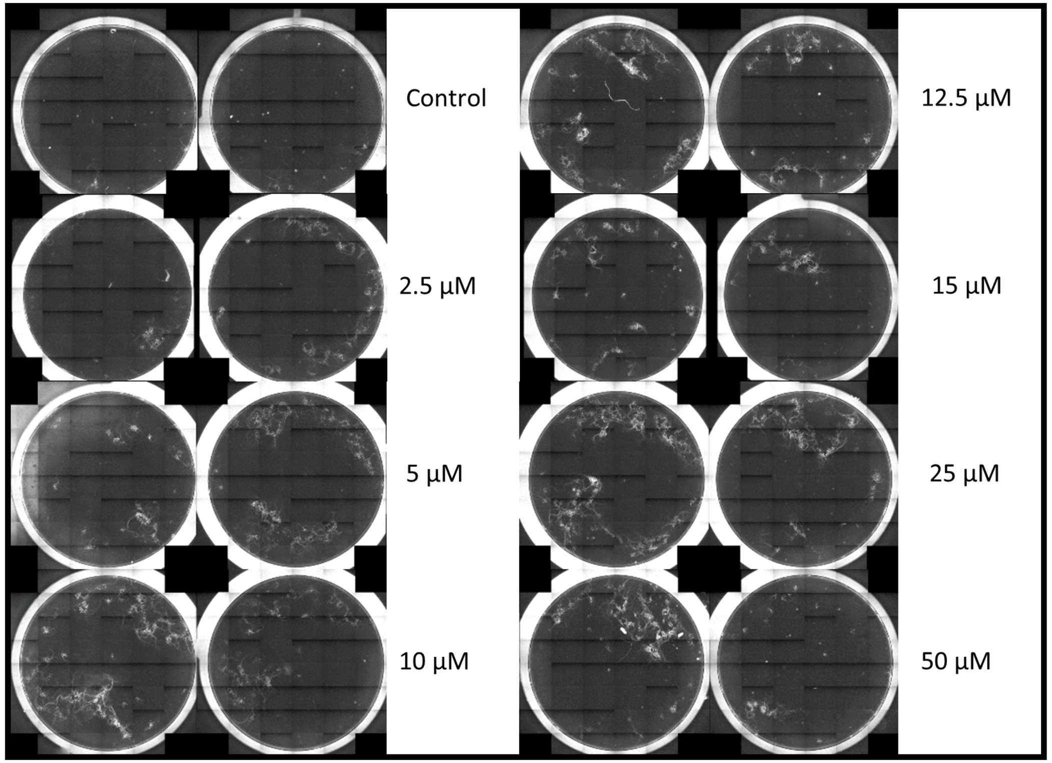

c

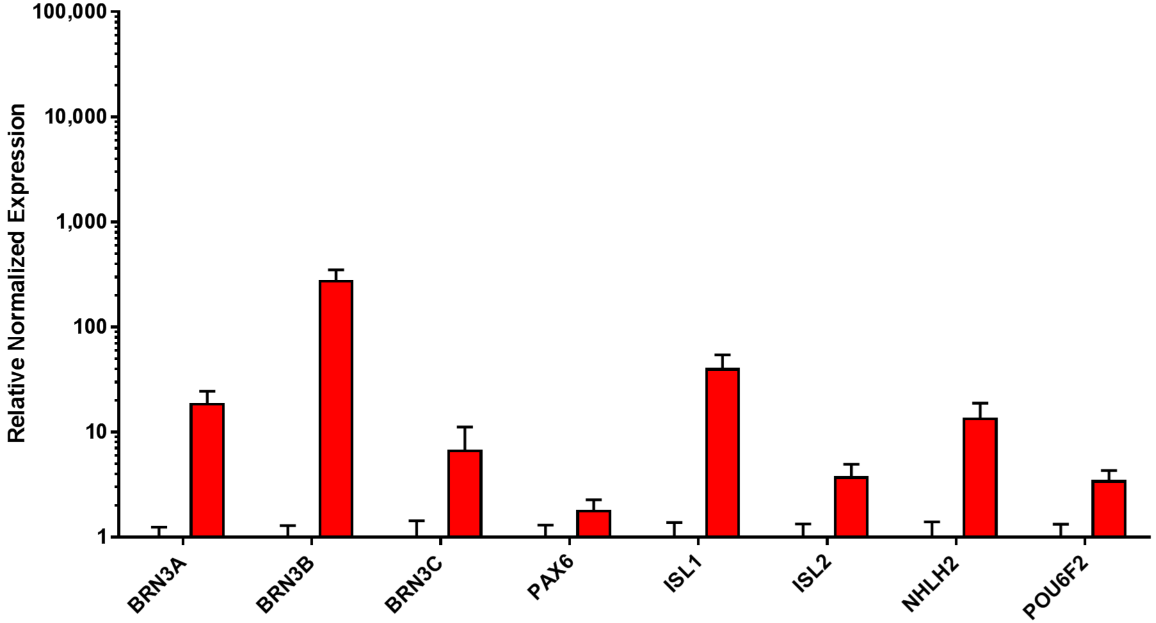

Figure S11

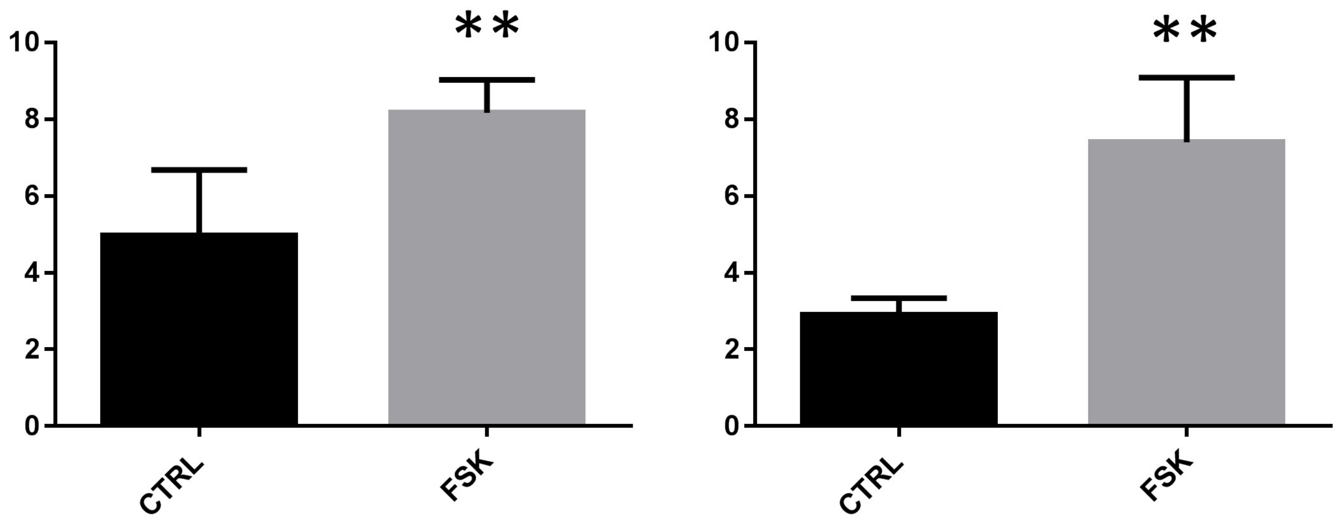

Figure S12

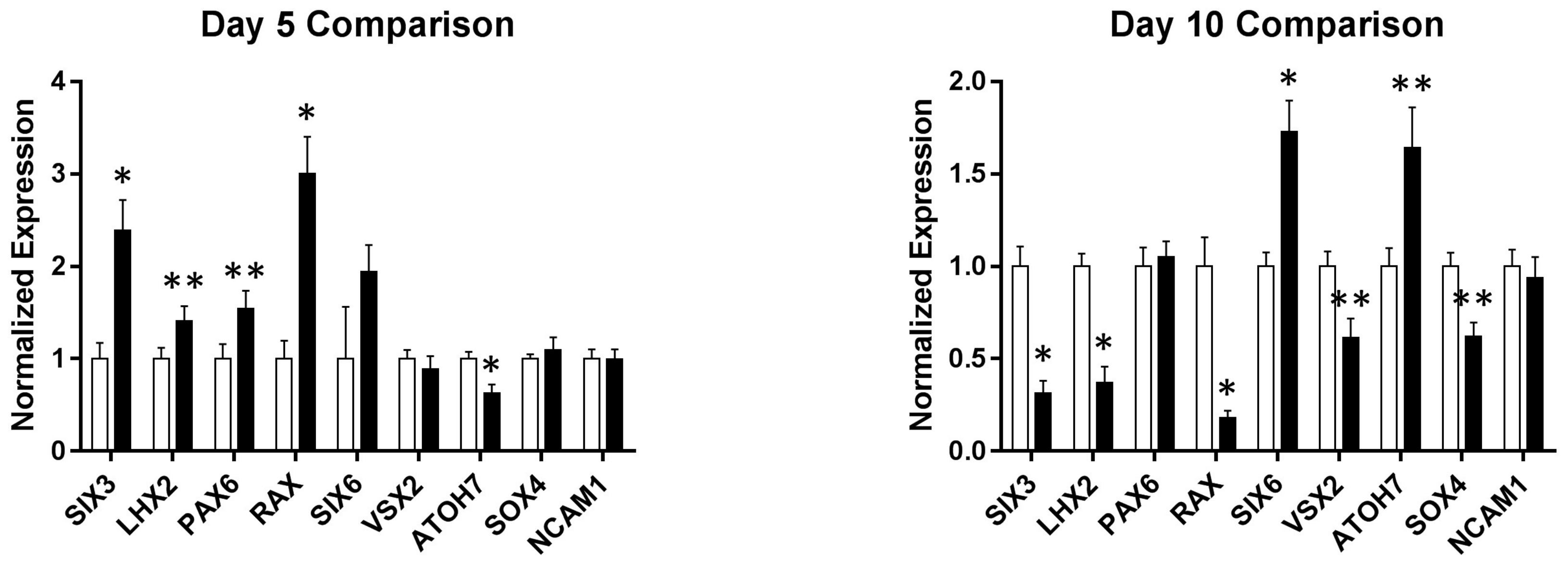
Day 5 Comparison
Normalized Expression
0
1
2
3
4
SIX3
LHX2
PAX6
RAX
SIX6
VSX2
ATOH7
SOX4
NCAM1
Day 10 Comparison
Normalized Expression
0.0
0.5
1.0
1.5
2.0
SIX3
LHX2
PAX6
RAX
SIX6
VSX2
ATOH7
SOX4
NCAM1

Supplementary Table 1 - Primer sequences for qPCR

Gene	Source	Forward primer	Reverse primer
ATOH7	PrimerBank:ID 38327523b1	CTGCCTTCGACCGCTTACG	CAGAGCCATGATGTAGCTCAG
BRN3A	PrimerBank:ID 110347448c1	GGGCAAGAGCCATCCTTTCAA	CTGTTCATCGTGTGGTACGTG
BRN3B	PrimerBank:ID 110347454b1	CAAGCAGCGACGCATCAAG	GGGTTTGAGCGCGATCATATT
BRN3C	PrimerBank:ID 225735566c1	CGACGCCACCTACCATACC	CGGGCATCACCGAGTGTTC
CABP5	PrimerBank:ID 329755246c1	ACAGCGGGAAAGACCACTG	CCCATCTCGGTCCTTATCGAA
CREBBP	Synnergren, 2007	GAGAGCAAGCAAACGGAGAG	AAGGGAGGCAAACAGGACA
CRX	PrimerBank:ID 189095267c1	GCCCCACTATTCTGTCAACG	GTCTGGGTACTGGGTCTTGG
ELAVL3	PrimerBank:ID 49355764c2	TCGAGTCCTGCAAGTTGGTTC	TGCATCATTGGGGTCAGAATAGT
GAD1	PrimerBank:ID 58331245c1	GCGGACCCCAATACCACTAAC	CACAAGGCGACTCTTCTCTTC
GAPDH	PrimerBank:ID 83641890b1	AAGGTGAAGGTCGGAGTCAAC	GGGGTCATTGATGGCAACAATA
GFAP	PrimerBank:ID 334688843c1	CTGCGGCTCGATCAACTCA	TCCAGCGACTCAATCTTCCTC
GS	PrimerBank:ID 260593717c2	TAAGGACCCTAACAAGCTGGT	CCGTTTACAGGTGTGCCTCAA
ISL1	PrimerBank:ID 115387113c2	TACGGGATCAAATGCGCCAA	CACACAGCGGAAACACTCGAT
ISL2	PrimerBank:ID 21956640c1	GTCAGATCCACGACCAGTTTATC	CCGCTTGCAGTAGGTCTTCC
LHX1	PrimerBank:ID 314122156c2	CATGCGCGTCATTCAGGTCT	GAGAAGGGACCATTGGGGAT
LHX2	PrimerBank:ID 30795195c1	ATGCTGTTCCACAGTCTGTCG	GCATGGTCGTCTCGGTGTC
MAP2	PrimerBank:ID 87578393c2	CGAAGCGCCAATGGATTCC	TGAACTATCCTTGCAGACACCT
mCherry	Geneious	GCCCGGCGCCTACAACGTCA	GCCCTCGGCGCGTTCGTACT
MITF	Meyer, 2009	TTCACGAGCGTCCTGTATGCAGAT	TTGCAAAGCAGGATCCATCAAGCC
NCAM1	PrimerBank:ID 336285437c1	GGCATTTACAAGTGTGTGGTTAC	TTGGCGCATTCTTGAACATGA
NEFH	PrimerBank:ID 196162718c1	GCAGTCCGAGGAGTGGTTC	CGCATAGCGTCTGTGTTCA
NEFL	PrimerBank:ID 197927150c1	ATGAGTTCCTTCAGCTACGAGC	CTGGGCATCAACGATCCAGA
NHLH2	PrimerBank:ID 161484630c1	GCAGCAGATTCGGACCATCC	ACGTGGTTGAGATAGGAGATGT
NRN1	PrimerBank:ID 34878876b1	CAAATAGCGTATCTGGTGCAGG	CTTGAAGACCGCATCGCACT
OPN4-1	PrimerBank:ID 121582618c1	ACCGCTACCTGGTAATCACAC	GCGTGAAGCTCATGTAGTCCC

OPN4-2	PrimerBank:ID 121582618c2	GGTACGCACACGTCCTGAC	GGGGTTGTGGATTGCAGAGG
PAX6	Meyer, 2009	AGTGAATCAGCTCGGTGGTGTCTT	TGCAGAATTCGGGAAATGTCGCAC
POU6F2	PrimerBank:ID 260436857c1	AGTAAGCCCTTGCTGTCAGTG	CCTGGGTCTGATAATGCTGGG
PRKCA	PrimerBank:ID 47157319c1	GTCCACAAGAGGTGCCATGAA	AAGGTGGGGCTTCCGTAAGT
PROX1	PrimerBank:ID 34147628c1	AAAGGACGGTAGGGACAGCAT	CCTTGGGGATTCATGGCACTAA
RAX-1	PrimerBank:ID 126116580c1	GGCCATCCTGGGGTTTACC	GGTCGAGGGGCTTCGTACT
RAX-2	PrimerBank:ID 126116580c2	AAGCCCCTCGACCCTACTG	CCGCCGATGCTTTTTCTTGG
RBPMS	PrimerBank:ID 57164972c1	AAACAGCCTGTAGGTTTTGTCA	GGAATTTCAGGATCGAAGCGG
RCVRN	PrimerBank:ID 56550117c1	CCAGAGCATCTACGCCAAGTT	CCGTCGAGGTTGGAATCGAAG
RHO	PrimerBank:ID 169808383c1	GTGCCCTTCTCCAATGCGA	TGAGGAAGTTGATGGGGAAGC
RPE65	Maruotti, 2013	TGCGTATGGACTTGGCTTGAATC	TCCTGCTCCTGGGCTCACC
SIX3	PrimerBank:ID 261878476c1	CTGCCCACCCTCAACTTCTC	GCAGGATCGACTCGTGTTTGT
SIX6	PrimerBank:ID 186910310c1	GCCCTCAACAAGAATGAGTCG	GCCTCCTGGTAGTGTGCTTC
SLC6A9	PrimerBank:ID 67782316c2	GATCAGCCCCATGTTCAAAGG	GTTGGAGGCGTCCAGTACAC
SNCG	PrimerBank:ID 4507113a1	TGAGCAGCGTCAACACTGTG	GAGGTGACCGCGATGTTCTC
SOX11	PrimerBank:ID 30581115c2	CGCCGACGACCTGATGTTC	CGAATCCAAATCCTTATCCACCA
SOX4	PrimerBank:ID 30179901c2	GACCTGCTCGACCTGAACC	CCGGGCTCGAAGTTAAAATCC
THY1	PrimerBank:ID 221136764b1	ATCGCTCTCCTGCTAACAGTC	CTCGTACTGGATGGGTGAACT
TUBB3	PrimerBank:ID 308235961c1	GGCCAAGGGTCACTACACG	GCAGTCGCAGTTTTCACACTC
VGLUT1 (SLC17A7)	PrimerBank:ID 221316691c2	CAGAGTTTTCGGCTTTGCTATTG	GCGACTCCGTTCTAAGGGTG
VSX2	PrimerBank:ID 156071534c2	TCATGGCGGAGTATGGGCT	TCCAGCGACTTTTTGTGCATC

Supplementary Table 2 – CRISPR Off-target

Chromosome	Start	End	Strand	MM	Target sequence	PAM	Position	Gene name
chr4	146640798	146640820	-	0	GCCAAGAGTCTTCTAAATGC	CGG	Exonic	POU4F2
chr13	66266202	66266224	+	4	**ATT**AAGA**A**TCTTCTAAATGC	AGG	Intergenic	TRIM60P19
chr10	22911490	22911512	+	4	**ATG**AAGAG**C**CTTCTAAATGC	AGG	Intergenic	ARMC3
chr2	9475196	9475218	+	4	G**GG**AA**CG**GTCTTCTAAATGC	AGG	Exonic	IAH1
chr4	105241992	105242014	-	4	**CAT**AAGAGTCTTCTA**C**ATGC	AGG	Exonic	TET2
chr1	181651533	181651555	-	4	G**GT**AAG**T**GTCTTCTAAATG**A**	AGG	Exonic	CACNA1E

Mismatch bases are in bold
